# Transcriptomic Insights Into Root Development and Overwintering Transcriptional Memory of *Brassica rapa* L. Grown in the Field

**DOI:** 10.3389/fpls.2022.900708

**Published:** 2022-07-22

**Authors:** Lijun Liu, Yuanyuan Pu, Zaoxia Niu, Junyan Wu, Yan Fang, Jun Xu, Fang Xu, Jinli Yue, Li Ma, Xuecai Li, Wancang Sun

**Affiliations:** ^1^State Key Laboratory of Aridland Crop Science, Gansu Agricultural University, Lanzhou, China; ^2^College of Agronomy, Gansu Agricultural University, Lanzhou, China; ^3^Shanghai OE Biotech Co., Ltd., Shanghai, China

**Keywords:** *Brassica rapa* L., transcriptomic, differentially expressed genes, root development, overwintering memory

## Abstract

As the only overwintering oil crop in the north area of China, living through winter is the primary feature of winter rapeseed. Roots are the only survival organ during prolonged cold exposure during winter to guarantee flowering in spring. However, little is known about its root development and overwintering memory mechanism. In this study, root collar tissues (including the shoot apical meristem) of three winter rapeseed varieties with different cold resistance, i.e., Longyou-7 (strong cold tolerance), Tianyou-4 (middle cold tolerance), and Lenox (cold-sensitive), were sampled in the pre-winter period (S1), overwintering periods (S2–S5), and re-greening stage (S6), and were used to identify the root development and overwintering memory mechanisms and seek candidate overwintering memory genes by measuring root collar diameter and RNA sequencing. The results showed that the S1–S2 stages were the significant developmental stages of the roots as root collar diameter increased slowly in the S3–S5 stages, and the roots developed fast in the strong cold resistance variety than in the weak cold resistance variety. Subsequently, the RNA-seq analysis revealed that a total of 37,905, 45,102, and 39,276 differentially expressed genes (DEGs), compared to the S1 stage, were identified in Longyou-7, Tianyou-4, and Lenox, respectively. The function enrichment analysis showed that most of the DEGs are significantly involved in phenylpropanoid biosynthesis, plant hormone signal transduction, MAPK signaling pathway, starch and sucrose metabolism, photosynthesis, amino sugar and nucleotide sugar metabolism, and spliceosome, ribosome, proteasome, and protein processing in endoplasmic reticulum pathways. Furthermore, the phenylpropanoid biosynthesis and plant hormone signal transduction pathways were related to the difference in root development of the three varieties, DEGs involved in photosynthesis and carbohydrate metabolism processes may participate in overwintering memory of Longyou-7 and Tianyou-4, and the spliceosome pathway may contribute to the super winter resistance of Longyou-7. The transcription factor enrichment analysis showed that the *WRKY* family made up the majority in different stages and may play an important regulatory role in root development and overwintering memory. These results provide a comprehensive insight into winter rapeseed's complex overwintering memory mechanisms. The identified candidate overwintering memory genes may also serve as important genetic resources for breeding to further improve the cold resistance of winter rapeseed.

## Introduction

As sessile organisms, plants have evolved complex regulatory mechanisms to cope with the changing ambient environment and to balance the growth and stress response processes to maximize survival/improve viability (Zhang et al., 2020). Cold stress is one of the major factors that influence plant growth and development, and limits crops' geographic distribution and productivity (Chinnusamy et al., 2010; Guo et al., 2018). Winter annual plants are generally sown in autumn and require experiencing prolonged cold exposure to acquire competence for flowers in the spring of the next year at a warm temperature (Amasino, 2010; Hepworth and Dean, 2015; O'Neill et al., 2019). Plants with an annual winter lifestyle prevent the transition from vegetative growth to floral development during winter cold periods by remembering the duration of exposure to winter cold; hence, plants can successfully overwinter in the seedling stage (Xu and Chong, 2018). Thus, how plants monitor seasonal cues and count winter cold time to avoid developmental transition during prolonged cold exposure in winter is a complex regulatory network.

Plants can develop a sophisticated transcriptional stress-memory mechanism under long-term stress, and stress memory may allow plants to successfully overcome a subsequent, more severe stress exposure (Olas et al., 2021). Transcriptional memory genes can be classified into four response patterns “+/+,” “–/–,” “+/–,” and “–/+.”The “+/+” and “–/–” patterns represent genes whose expression is upregulated and downregulated, respectively, by early stress relative to expression levels of pre-stressed plants, whereby the expression level was higher or lower during subsequent stresses relative to the level of early stress. The “+/–” and “–/+” patterns represent genes whose expression is upregulated and downregulated, respectively, by early stress compared to the pre-stressed condition, whereby the expression level was lower or higher during subsequent stresses relative to the level of early stress (Ding et al., 2013). Previous studies on overwintering memory mainly focused on vernalization-mediated flowering and revealed that the genetic/epigenetic regulation mechanisms of “memory of winter cold” are different between monocotyledons and dicotyledons (Bouché et al., 2017; Xu and Chong, 2018; Luo and He, 2020). In Arabidopsis of dicotyledons, *FLOWERING LOCUS C* (*FLC*) is the key flowering repressor to count winter dosage and regulate flowering time in warm spring (Sheldon et al., 2000; Xu and Chong, 2018). As a MADS-box transcription factor, the upregulation of *FLC* inhibits the expression of two key floral regulatory factors, *FT* (*FLOWERING LOCUS T*) and *SOC1* (*SUPPRESSOR OF OVEREXPRESSION OF CO 1*) (Michaels et al., 2005; Sheldon et al., 2006). During prolonged cold exposure in winter, the expression of *FLC* is inhibited by the PRC2 complex by deposition of H3K27me3 modification in the *FLC* nucleation zone (Zhu et al., 2015), and the silenced state is maintained until warm spring to ensure flowering (Hepworth and Dean, 2015; Zhu et al., 2015; Whittaker and Dean, 2017). In temperate grasses of monocotyledons, including *Brachypodium*, wheat, and barley, *VRN1* is the central promoter of the vernalization-induced flowering pathway to measure the length of cold exposure for floral initiation from vegetative growth (Oliver et al., 2009; Xu and Chong, 2018). *VRN1* encodes an APETALA1 (AP1)-like MADS-box transcription factor (Yan et al., 2003), whose expression is induced by winter cold and maintained at a high level by increased deposit of H3K4me3 and H3K36me3 modifications in the first intron and promoter regions by the Trithorax group complex in warm conditions to accelerate flowering (Simon and Tamkun, 2002; Oliver et al., 2009; Diallo et al., 2012; Chittock et al., 2017). However, whether there are other overwintering transcriptional memory mechanisms and candidate overwintering memory genes has not been reported. Moreover, winter memory in dicotyledonous plants is well-studied in the model plant *Arabidopsis thaliana* (Hepworth et al., 2018; Luo et al., 2020). Thus, research on winter memory of other species such as winter rapeseed (*Brassica rapa* L.) will expand our understanding of the similarities and differences in winter memory between monocotyledonous and dicotyledonous plants. Although the genetic basis of flowering time in *Brassica napus* has been studied in recent years (O'Neill et al., 2019; Matar et al., 2021), research on the winter memory mechanism in winter rapeseed is still lacking.

Winter rapeseed is the only overwintering cruciferous oilseed crop in the north area of China. Therefore, planting cold-resistant varieties can increase the sowing area of oil crops and guarantee the supply of edible oil (Jian et al., 2020). Winter turnip rape (*Brassica rapa* L.) is sown in the middle of August in the northwest of China. It needs to experience a long overwintering period, so living through the winter is the primary feature. During prolonged cold exposure in winter, the aboveground part of winter turnip rape begins to wither in the eight to nine true leaf stages, while the roots, including the shoot apical meristem, still survive to overwinter. In warm spring, the aboveground parts of winter turnip rape start to turn green (Liu et al., 2013). Therefore, it is important to research on the overwinter mechanism of winter rapeseed roots (including shoot apical meristem), which are the only living part during winter.

Until now, many studies have been focused on the morphological structures, physiological and biochemical levels, and molecular biology of the cold tolerance of *Brassica rapa* L. (Yang et al., 2014). Recent studies have reported that cold-resistant varieties of *Brassica rapa* L. exhibit more significant changes in metabolic activity-related proteins at low temperature than cold-sensitive varieties by transcriptomics, proteomics, and small RNA sequencing analyses (Zeng et al., 2018a,b; Ma et al., 2019; Niu et al., 2021). Some previous studies have also investigated matter transport, photosynthetic characteristics, fluorescence dynamics (Xu et al., 2020a), endogenous hormones (Xu et al., 2020b), and the morphological and physiological mechanisms (Niu et al., 2021) under winter cold exposure of different winter rapeseed varieties with contrasting overwintering abilities grown in the field. The results showed that photosynthetic capacity and malondialdehyde (MDA) and indole-3-acetic acid (IAA) content were decreased, and that soluble sugar and soluble protein content and peroxidase (SOD, POD) activity were increased in cold-resistant variety as compared to cold-sensitive varieties grown in the field. However, fewer studies have addressed the molecular mechanism of overwintering memory and overwinter memory genes in *Brassica rapa* L. Thus, research on the overwinter memory mechanism would greatly improve cold resistance in *Brassica rapa* L. The present study explores the overwintering memory mechanism and identifies overwinter memory genes in three winter rapeseed varieties with different cold resistance at the transcriptome level. As roots are the critical part and only living organ of winter rapeseeds for overwinter survival, root collar tissues (including the shoot apical meristem) of Longyou-7, Tianyou-4, and Lenox were sampled in the pre-winter period (S1 stage), overwintering period (S2–S5 stages), and re-greening stage (S6 stage) and used to perform RNA-Seq analyses. This study provides an insight into complex overwintering memory mechanisms in winter rapeseed (*Brassica rapa* L.). Furthermore, the identified candidate overwinter memory genes also serve as important genetic resources for breeding cold resistance varieties.

## Materials and Methods

### Plant Materials, Field Trails, and Sample Collection

Three winter turnip rape varieties with contrasting overwintering abilities from Gansu Agricultural University were used in this study: Longyou-7 (L7, strong cold tolerance, with a considerable higher overwinter survival rate of 99% in Shangchuan), Tianyou-4 (T4, middle cold tolerance, with a relative lower overwinter survival rate of 78% in Shangchuan), and Lenox (cold-sensitive, with extreme low overwinter survival rate in Shangchuan, close to zero). Winter turnip rapeseeds were sown and maintained outside in the field at Gansu Research Center of Rapeseed Engineering and Technology located in Shangchuan town, Yongdeng county, Lanzhou city, Gansu province (longitude: 103.67°E; latitude: 36.05°N; altitude: 2,180 m). Samples were collected periodically from fall to spring, and root collar diameter was measured at the same time, with specific sampling dates being 9 October (L7_S1, T4_S1, and Lenox_S1 as control), 2 November (L7_S2, T4_S2, and Lenox_S2), 24 November (L7_S3, T4_S3, and Lenox_S3), 15 December 2019 (L7_S4, T4_S4, and Lenox_S4), 4 January (L7_S5, T4_S5, and Lenox_S5) and 25 April 2020 (L7_S6 and T4_S6). The first sampling day with a minimum air temperature of 4°C occurred on 9 October 2019, and the mean minimum air temperature from 2 November 2019 to 4 January 2020 was −13°C, whereas the re-greening sampling date with a minimum air temperature of −1°C occurred on 25 April 2020. With each sampling date, plants were collected simultaneously in the field (between 10:30 and 11:30 a.m.) to eliminate the effect of circadian clock. As roots are critical tissues and the only living organ of winter rapeseeds for overwinter survival, the same proportion of root collar tissues (a 5-mm section below the cotyledon nodes), including the shoot apical meristem (a 3-mm section above the crown base) in six sampling stages was selected for RNA-Seq analyses. Each sample was collected from more than three plants, immediately frozen in liquid nitrogen, and stored at −80°C until analysis. Three biological replicates of each sampling point were performed for this study except for L7_S4 and L7_S6 that had two replicates as the other sample was contaminated by plant viruses and Lenox_S6 that had no sample because only little plants of Lenox can survive to overwinter or the overwintering plants were contaminated.

### RNA Extraction, cDNA Library Construction, and Illumina Sequencing

The total RNA of each biological replicate was extracted using an RNAprep Pure plant kit (TianGen Biotech., Ltd., Beijing, China, DP441) according to the manufacturer's protocol, and genomic DNA was digested using RNase-free DNase (Promega, United States) for each sample. Then, the RNA purity and concentration of all the samples were measured using a NanoDrop 2000 spectrophotometer (Thermo Fisher Scientific, Wilmington, DE, United States), and RNA integrity was evaluated using the Agilent 2100 Bioanalyzer (Agilent Technologies, Santa Clara, CA, United States). For RNA-seq analysis, equal amounts of RNA extracted from each sample were used to construct a cDNA library using a TruSeq Stranded mRNA LT Sample Prep Kit (Illumina, San Diego, CA, United States) according to the manufacturer's instructions. Briefly, mRNA was enriched with oligo (dT)-magnetic beads and broken into short fragments (≈200 bp) with a fragmentation buffer. The short mRNA fragments were used as templates to synthesize the first-strand cDNA with random hexamer primers. Then, double-stranded cDNA fragments were synthesized by the addition of buffer, dNTPs, RNase H, and DNA polymerase I that were purified using a QiaQuick PCR extraction kit (Qiagen). The purified double-stranded cDNA fragments were then terminally repaired. Finally, the repaired fragments were added with poly A and ligated to adaptor sequences.

Subsequently, fragment size selection was carried out, and cDNA libraries were constructed by PCR amplification with the ligated products. The constructed libraries underwent quality checks using the Agilent 2100 Bioanalyzer. Finally, the qualified cDNA libraries were sequenced on an Illumina HiSeq X Ten sequencer to generate 150-bp paired-end reads by OE Biotech Co., Ltd. (Shanghai, China).

### Bioinformatics Analysis of RNA-Seq

After sequencing, raw reads for each sample were obtained (NCBI accession number: PRJNA811760). First, raw reads in fastq format were processed using the Trimmomatic software (Bolger et al., 2014), and sequencing adapters, poly-N, and low-quality reads were removed to obtain clean reads for subsequent analyses. Then, filtered clean reads were independently aligned to the *Brassica rapa* genome survey version 3.01 sequence (Zhang et al., 2018) obtained from National Center for Biotechnology Information (https://ftp.ncbi.nlm.nih.gov/genomes/all/GCF/000/309/985/GCF_000309985.2_CAAS_Brap_v3.01/GCF_000309985.2_CAAS_Brap_v3.01_genomic.fna.gz) using HISAT2 (Kim et al., 2015). Fragments per kilobase of transcript per million fragments sequenced (FPKM) (Roberts et al., 2011) was used as the measurement unit to estimate the abundance of gene transcripts and was calculated using Cufflinks (Trapnell et al., 2010). HTSeq (Anders et al., 2015) was used to calculate the read counts of each gene. Differential expression analysis was carried out using the DESeq (2012) R package (Anders and Huber, 2012). False discovery rate (FDR) > 0.001, *P* value < 0.05, and fold change > 2 or fold change < 0.5 were set as the threshold to identify significance differentially expressed genes (DEGs). To show the gene expression pattern among different samples, a hierarchical cluster analysis of DEGs was performed using the T-MeV 4.9.0 software (Howe et al., 2011). Principal component analysis was performed to calculate the distance between different samples using the DESeq (2012) R package/clustering method (Anders and Huber, 2012). Functional annotation of DEGs was conducted by GO enrichment and KEGG (Kanehisa et al., 2008) pathway enrichment analyses using R based on hypergeometric distribution. Short Time-series Expression Miner (STEM) was used to analyze different gene expression trends and identify candidate overwintering memory genes (Ernst and Bar-Joseph, 2006). Alternatively, splicing events of differentially regulated transcript isoforms or exons were identified using the ASprofile software (Florea et al., 2013). SNPs and InDels were evaluated using SAMtools (Li et al., 2009) and BCFtools (Li, 2011), respectively.

### Verification by qRT-PCR

Real-time PCR amplification was carried out on a QuantStudio 5 real-time PCR system (ABI, United States) using SYBR Green I (Takala, RR820A, Dalian, China) to validate the transcriptome sequencing results. Two micrograms of total RNA were used to synthesize the first strand of cDNA following the protocol of the PrimeScript™ RT Master Mix kit (TaKaRa, RR036A, Dalian, China). Eleven DEGs were selected for qRT-PCR analysis, and the specific primer sequences are shown in Supplementary Table 15. The relative expression level of each gene was calculated using the 2^−Δ*ΔCt*^ method (Livak and Schmittgen, 2001) vs. the reference gene *Actin*. Each gene in a single sample was tested for three independent technical repeats and three biological replicates to calculate mean values and standard errors.

## Results

### Root Collar Diameter Analysis of the Three Winter Turnip Rape Varieties With Contrasting Overwintering Abilities

**Figure 1:**
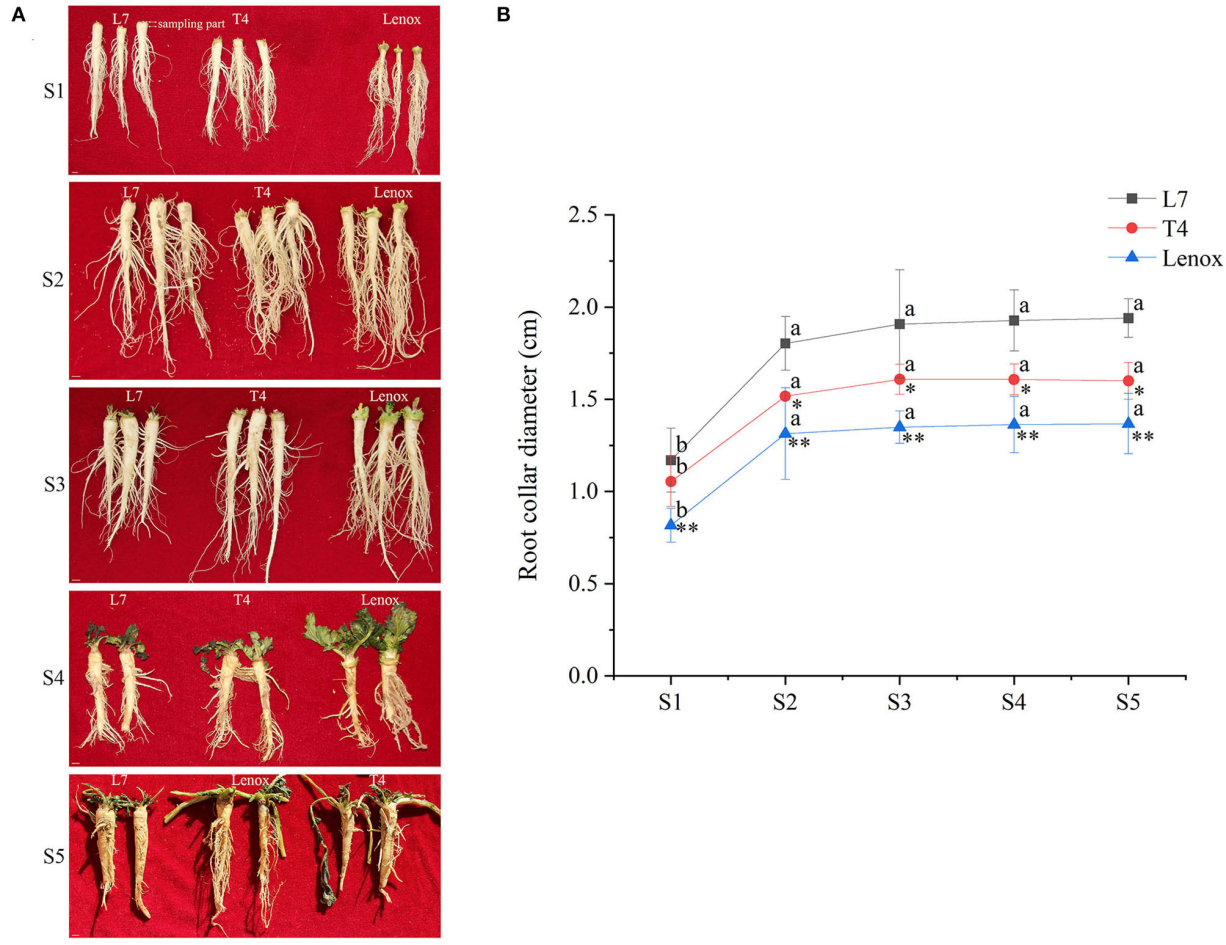
Morphological characteristics of roots of three winter turnip rape varieties. **(A)** Phenotype of roots during five sampling stages. S1, S2, S3, S4, and S5 represent the pre-winter period (S1) and overwintering periods (S2–S5), respectively. The scale bar is 1 cm. **(B)** Root collar diameter in winter. Samples (*n* ≥ 5) were measured every sampling date. Letters indicate significant differences between different stages of each variety compared to the S1 stage, and asterisks indicate significant differences between different varieties compared to Longyou-7 (L7) in every stage (*p < 0.05, **p < 0.01, Duncan).

As roots are the only living organ of winter rapeseeds for overwinter survival, and root collar diameter is always used as a morphological indicator to identify the overwintering ability of winter rapeseeds, the root collar diameter of Longyou-7, Tianyou-4, and Lenox with contrasting overwintering abilities was measured to evaluate the developmental features of roots in each of the sampling stages (S1-S5 stages) under field conditions from autumn to winter. The results showed that in the whole winter period, the root collar diameter of Longyou-7 was larger than that of Tianyou-4, but that Tianyou-4 was larger than Lenox (Figure 1). This result was consistent with the overwintering ability of the three varieties. During the S1 stage, the root collar diameter of Longyou-7, Tianyou-4, and Lenox was 1.17, 1.05, and.82 cm, respectively. In the S2 stage, the root collar diameter of Longyou-7 increased to 1.8 cm, and the root collar diameter of Tianyou-4 and Lenox reached 1.52 and 1.31 cm, respectively. This stage showed a significant increase in the root growth of each variety. In the S3–S5 stages, the root collar diameter of the three varieties increased slowly. It is conceivable that the S2 stage may play an important role in determining root growth and overwintering ability.

### RNA-Seq Analysis of Three Winter Turnip Rape Varieties With Contrasting Overwintering Abilities

To explore the molecular mechanism of the growth and overwintering ability differences, RNA-Seq analyses of root collars were conducted to compare the global transcriptome differences of Longyou-7, Tianyou-4, and Lenox in the pre-winter period (S1), overwintering periods (S2–S5), and re-greening (S6) stage (six sampling periods from each cultivar except for Lenox with five sampling periods as Lenox cannot re-green or overwinter). As shown in Supplementary Table 1, a total of 345.19 Gb of clean data (average ≈6.76 Gb clean data for each sample) were generated from 51 libraries, and the percentage of Q30 bases for all the libraries ranges from 92.37 to 95.06%, while the GC content of each treatment was almost 48%. The clean reads were mapped to the *Brassica rapa* reference genome (Supplementary Table 1), multiple mapped reads were removed, and uniquely mapped reads were used for subsequent analysis. The percentage of uniquely mapped reads ranged from 78.59 to 87.99%. The uniquely mapped reads were processed with the cufflinks software to calculate the normalized abundance of gene transcripts as fragments per kilobase of transcript per million fragments sequenced (FPKM). The correlation coefficient between biological replications of different sampling stages was used to evaluate the reliability of the samples and was calculated based on the FPKM of each sample. The results showed that the correlation coefficients between the samples were close to 1, indicating a high similarity of the biological replications.

### Global Transcriptome Comparison of the Three Varieties in Different Stages

**Figure 2:**
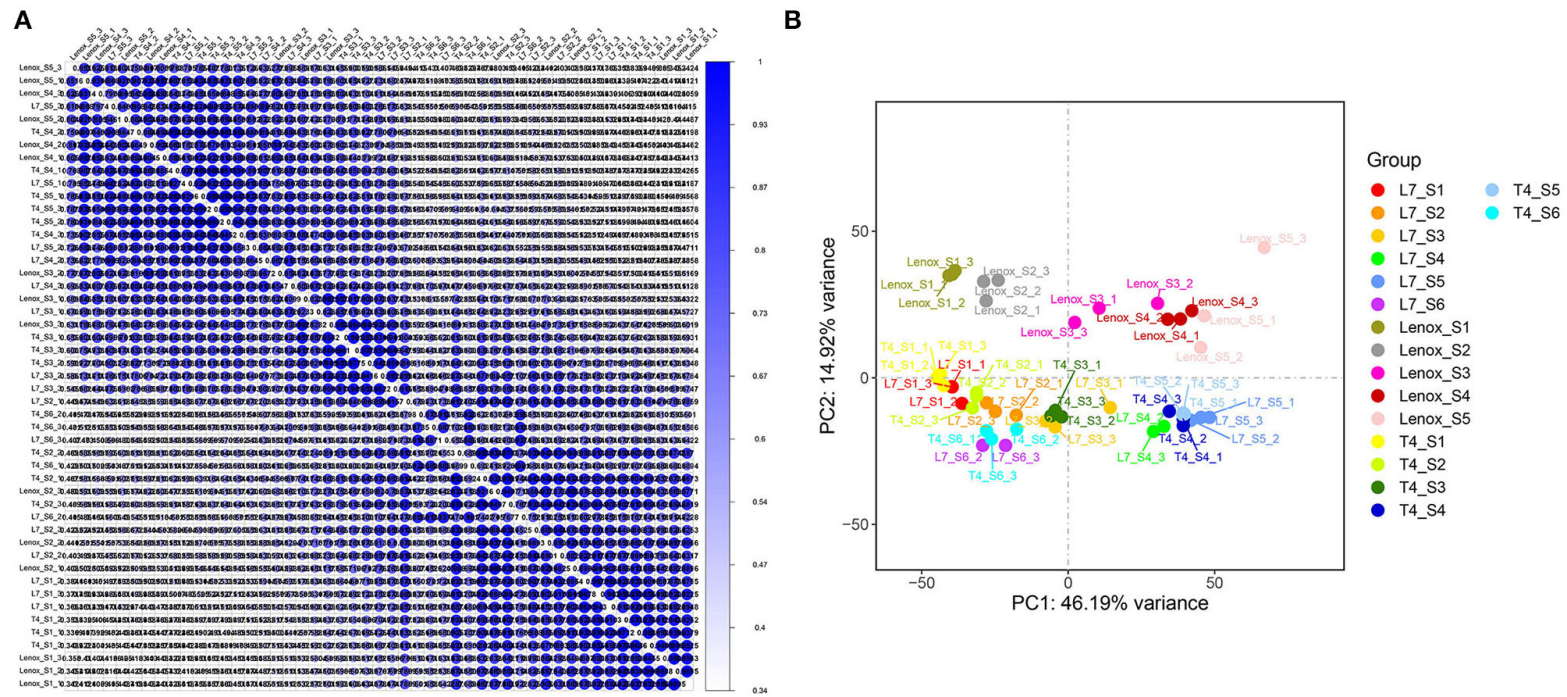
Correlation analyses of RNA-Seq data in different stages of the three varieties. **(A)** Spearman correlation coefficient (SCC) analysis of different stages in Longyou-7 (L7), Tianyou-4 (T4), and Lenox. **(B)** Principal component analysis (PCA) analysis showing clustering of transcriptome s changes in different stages of Longyou-7 (L7_S1–L7_S6), Tianyou-4 (T4_S1–T4_S6), and Lenox (Lenox_S1–Lenox_S5).

To better evaluate the global dynamic changes in the transcriptome during different developmental stages in the three varieties, principal component analysis (PCA) and hierarchical clustering analysis were performed based on the average FPKM values of all the 41,405 identified unigenes (Figure 2). Both analyses showed a higher correlation between the same developmental stage and substantial diversities with different stages among the three varieties. The PCA (Figure 2A) results demonstrated that: (i) biological replicates in each group were clustered together, (ii) principal component 1 separated samples from different stages, the S1 and S2 stages showed distant correlation in each cultivar, and the clustering of the S3 stage from the three varieties was different, as the S3 stage of Longyou-7 and Tianyou-4 tended toward the S2 stage, whereas the S3 stage of Lenox exhibited a closer correlation with the S4 stage, the S4 and S5 stages showed very tight clustering in each cultivar, whereas the S6 stage of the Longyou-7 and Tianyou-4 varieties was grouped together with the S2 stage, indicating notable transcriptome shift in different stages, (iii) principal component 2 separated Lenox from Longyou-7 and Tianyou-4, and samples from the Longyou-7 and Tianyou-4 varieties in all the stages clustered tightly, implying a higher similarity in transcriptional programs of the two varieties and innate differences of Lenox compared to the other two varieties. The results implied significant differences in the transcriptome of different stages among the three varieties. Furthermore, the transcriptome difference in the S2 and S3 stages may determine key genes related to the root development and overwintering abilities of the three varieties.

### Identification and Functional Annotation of DEGs in Different Stages Versus S1 Stage of the Three Winter Turnip Rape Varieties

**Figure 3:**
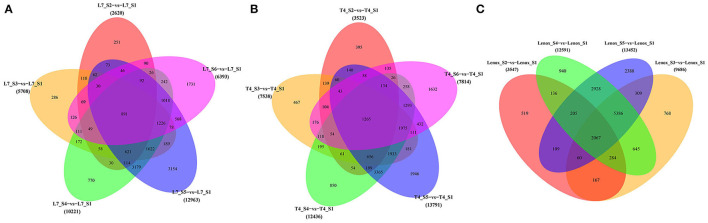
Venn diagrams of differently expressed genes on different stages of **(A)** Longyou-7 (L7), **(B)** Tianyou-4 (T4), and **(C)** Lenox.

To investigate the differences in transcription levels that characterize the different developmental stages in each of the three varieties with contrasting overwintering abilities, FPKM was used to calculate the expression levels for screening DEGs. Based on the criterion of adjusted *p*-value < 0.05 and |log2FC| > 1, we identified a total of 37,905 (with 18,238 upregulated and 19,667 downregulated genes), 45,102 (with 21,603 upregulated and 23,499 downregulated genes), and 39,276 (with 17,618 upregulated and 21,658 downregulated genes) DEGs compared to the S1 stage in Longyou-7, Tianyou-4, and Lenox, respectively (Supplementary Figure 2, Supplementary Table 1). The overall transcriptome changes showed a high similarity among the three varieties. Venn diagrams were established to identify stage-specific and commonly expressed DEGs in different pairwise comparisons of the three varieties (Figure 3). In Longyou-7, 891 DEGs were commonly identified up- or down-regulated in the five pairwise comparisons, and 251, 286, 770, 3,154, and 1,731 DEGs were uniquely expressed in the five stages (S2–S6), respectively. In Tianyou-4, 1,265 DEGs were identified commonly expressed in the five pairwise comparisons, and 395, 467, 850, 1,946, and 1,632 DEGs were specifically identified in the five stages (S2–S6), respectively. In Lenox, the number of commonly expressed DEGs was 2,067 in the four pairwise comparisons, with 519, 768, 940, and 2,388 uniquely expressed DEGs in the four stages (S2–S5), respectively. Overall, the lowest number of stage-specific expressed DEGs was identified in the S2 stages (251 in Longyou-7, 395 in Tianyou-4, and 519 in Lenox). In contrast, in the S3–S5 stages, the number of stage-specific DEGs was gradually increased, and the S5 stage showed the largest number of DEGs in the three varieties (3,154 in Longyou-7, 1,946 in Tianyou-4, and 2,388 in Lenox). In the S6 stage of Longyou-7 and Tianyou-4, stage-specific DEGs were decreased compared to the S5 stage. To sum up, the variable number of specifically expressed DEGs in each stage among the three varieties may reflect that each stage has its independent role in developing and overwintering programs. The S2 stage may play an important role in root development, and the S3–S5 stages may affect the overwintering ability, especially in the S5 stage, whereas DEGs in the S6 stage may be related to the flowering process after re-greening.

**Figure 4:**
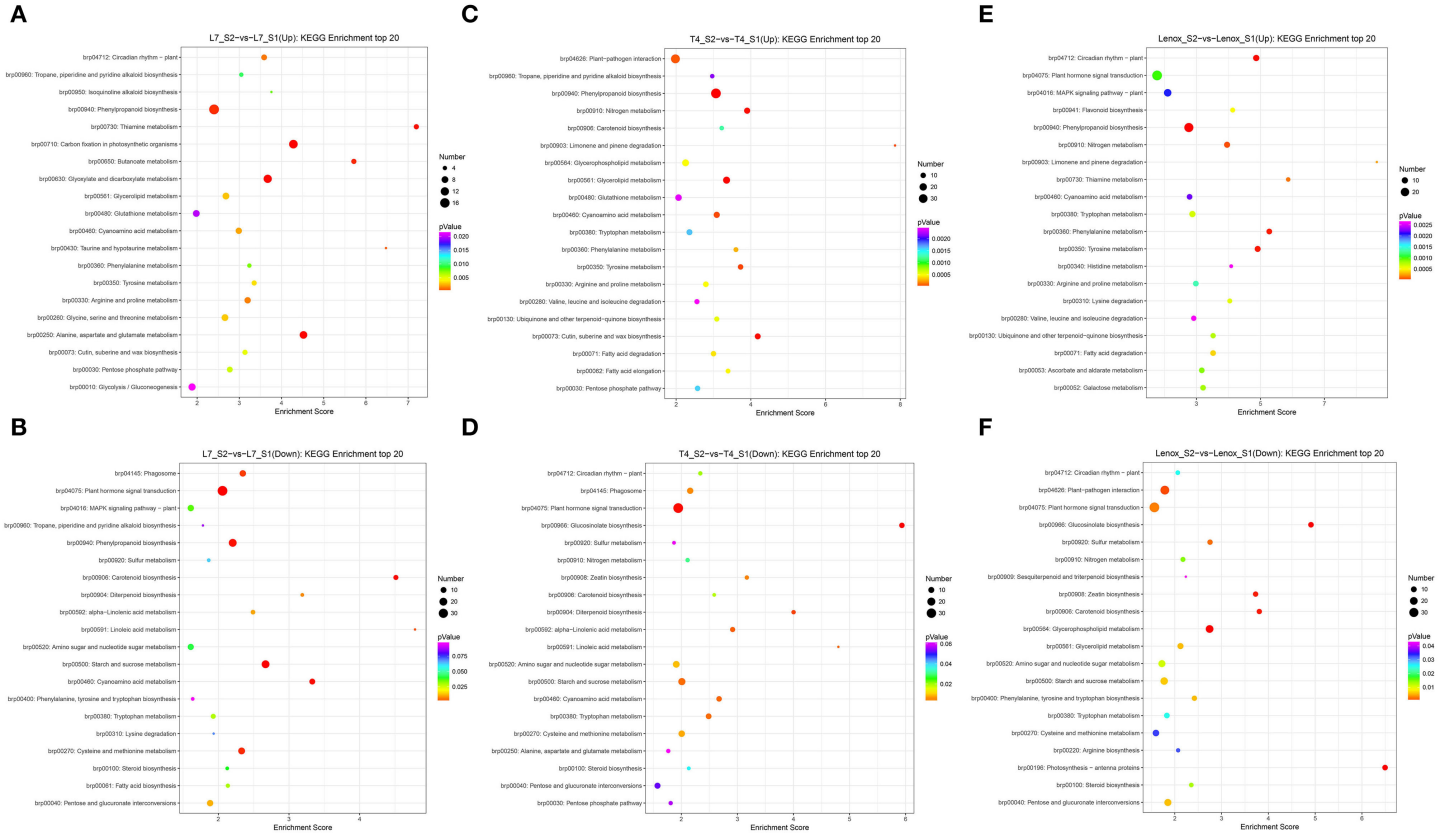
KEGG enrichment of differently expressed genes in the S2 stages of **(A,B)** Longyou-7 (L7), **(C,D)** Tianyou-4 (T4), and **(E,F)** Lenox.

To further classify the functions of the DEGs in the three varieties in different stages, a GO analysis was performed to divide the DEGs into three categories, i.e., biological process (BP), molecular function (MF), and cellular component (CC) (Supplementary Tables 2, 3). Among all the pairwise comparisons, “cellular process,” “metabolic process, “single-organism process,” “response to stimulus, “regulation of biological process,” “biological regulation,” and “developmental process” were dominant in “biological process,” “cell,” “cell part,” “organelle,” and “membrane” were dominant in “cellular component,” “binding,” and “catalytic activity” were dominant in “molecular function.” It is noticeable that more DEGs were functionally classified into the biological process than the cellular component and molecular function. A KEGG pathway analysis was also conducted to explore the potential function of DEGs in the three varieties in different stages (Supplementary Tables 4, 5). The DEGs in all the pairwise comparisons were significantly enriched in “carbohydrate metabolism,” “amino acid metabolism,” “lipid metabolism,” “energy metabolism,” “signal transduction,” “folding, sorting, and degradation,” and “translation.” The top 20 of the KEGG pathway analysis in each pairwise comparison showed that (Supplementary Table 6) in the S2 stage (Figure 4), phenylpropanoid biosynthesis (brp00940) was enriched largest by upregulated DEGs, and plant hormone signal transduction (brp04075) by downregulated DEGs in Longyou-7 and Tianyou-4. In contrast, plant hormone signal transduction (brp04075) was significantly enriched by both up- and downregulated DEGs in Lenox. Thus, the phenylpropanoid biosynthesis and plant hormone signal transduction pathways may influence the root development of the three varieties. In the S3 stage, the upregulated DEGs were mainly involved in plant-pathogen interaction (brp04626) and MAPK signaling pathway-plant (brp04016) in Longyou-7, the plant hormone signal transduction (brp04075) and MAPK signaling pathway-plant (brp04016) in Tianyou-4, and the plant hormone signal transduction (brp04075) and protein processing in the endoplasmic reticulum (brp04141) in Lenox, whereas the downregulated DEGs were mostly involved in starch and sucrose metabolism (brp00500), cysteine and methionine metabolism (brp00270), and amino sugar and nucleotide sugar metabolism (brp00520) in the three varieties. In the S4–S5 stages, spliceosome (brp03040) was enriched largest by upregulated DEGs and ribosome (brp03010) by downregulated DEGs in three varieties, except that plant hormone signal transduction (brp04075) was significantly enriched by the upregulated DEGs in Lenox. In the S6 stage, the upregulated DEGs were mainly involved in plant hormone signal transduction (brp04075), MAPK signaling pathway-plant (brp04016), and phenylpropanoid biosynthesis (brp00940) in Longyou-7 and Tianyou-4. The downregulated DEGs were mostly involved in ribosome (brp03010) and starch and sucrose metabolism (brp00500), and amino sugar and nucleotide sugar metabolism (brp00520). To sum up, the DEGs involved in different metabolic pathways, hormone signaling, and genetic information processing were related to the contrasting overwintering ability of the three varieties.

As the S2 stage was found important for root development (Figure 1), the specific and commonly expressed DEGs in the three varieties were also identified in the S2 stage compared to the S1 stage (Supplementary Figure 3A). A total of 922 DEGs were identified commonly expressed in the three varieties, with 1,007, 1,311, and 1,502 uniquely expressed DEGs in Longyou-7, Tianyou-4, and Lenox, respectively. The 1,007 uniquely expressed DEGs in Longyou-7 would decipher the faster root development and super winter resistance of Longyou-7 compared to the other two varieties. Except for the 922 commonly expressed DEGs in the three varieties, there were also 429 DEGs commonly expressed in Longyou-7 and Tianyou-4, which may be the main regulatory genes that account for the higher overwintering ability of Longyou-7 and Tianyou-4 than that of Lenox. The GO analysis indicated that cellular response to potassium ion starvation (GO:0051365, BP), apoplast (GO:0048046, CC), and chloride transport (GO:0006821, BP) were the significantly enriched top three items in the 429 commonly expressed DEGs in Longyou-7 and Tianyou-4 but not in Lenox. However, sinapyl alcohol dehydrogenase activity (GO:0052747, MF), cinnamyl-alcohol dehydrogenase activity (GO:0045551, MF), and chloroplast thylakoid membrane (GO:0009535, CC) were the markedly enriched top three items in the 1,007 uniquely expressed DEGs in Longyou-7. The KEGG pathway analysis revealed that metabolic pathways were preponderant. Among the 429 commonly expressed DEGs in Longyou-7 and Tianyou-4, 86 were annotated to the KEGG pathway and enriched in nine KEGG pathways (*p*-value ≤ 0.05, Supplementary Table 7). Eleven DEGs were enriched in phenylpropanoid biosynthesis (brp00940, *p*-value: 0.00022), six were involved in cyanoamino acid metabolism (brp00460, *p*-value: 0.00051), and four in cutin, suberine, and wax biosynthesis (brp00073, *p*-value: 0.005). Of the 1,007 uniquely expressed DEGs in Longyou-7, 181 were annotated to the KEGG pathway and enriched in eight KEGG pathways (*p*-value ≤ 0.05, Supplementary Table 7). Nine DEGs were enriched in glyoxylate and dicarboxylate metabolism (brp00630, *p*-value: 0.003), two were enriched in betalain biosynthesis (brp00965, *p*-value: 0.011), thirteen were enriched in phenylpropanoid biosynthesis (brp00940, *p*-value: 0.013), and twenty were involved in plant hormone signal transduction (brp04075, *p*-value: 0.013). These pivotal KEGG pathways, especially the phenylpropanoid biosynthesis and plant hormone signal transduction pathways, provide us with crucial information to seek the different mechanisms of root development processes in the three winter rapeseed varieties.

The specific and commonly expressed DEGs in the S3–S5 stages that may be related to the contrasting overwintering ability were also identified in the three varieties (Supplementary Figures 3B–D). A total of 3,656, 7,237, and 8,627 DEGs were commonly expressed in the three varieties in the S3–S5 stages, respectively. In the S3–S5 stages, 856, 1,193, and 1,760 DEGs were uniquely expressed in Longyou-7, and 1,240, 1,725, and 1,919 DEGs were uniquely expressed in Tianyou-4, but more DEGs were uniquely expressed in Lenox with 3,312, 2,421, and 1,936 DEGs in these stages. Besides, a total of 560, 1,166, and 1,466 DEGs were commonly expressed in Longyou-7 and Tianyou-4 but not in Lenox in the S3–S5 stages. The GO analysis (Supplementary Table 7) demonstrated the commonly expressed DEGs in S3–S5 stages in Longyou-7 and Tianyou-4 but not in Lenox. The significantly enriched top three GO terms were “defense response to bacterium” (GO:0042742, BP, 24 DEGs), “chloroplast thylakoid” (GO:0009534, CC, 14 DEGs), and “proline catabolic process to glutamate” (GO:0010133, BP, three DEGs) in the S3 stage, “proteasome regulatory particle, base subcomplex” (GO:0008540, CC, seven DEGs), “anthocyanin-containing compound biosynthetic process” (GO:0009718, BP, eight DEGs), and “hydrogen sulfide biosynthetic process”(GO:0070814, BP, five DEGs) in the S4 stage, and “chloroplast thylakoid” (GO:0009534, CC, 30 DEGs), “chloroplast thylakoid membrane” (GO:0009535, CC, 47 DEGs), and “response to karrikin” (GO:0080167, BP, 25 DEGs) in the S5 stage. In the Longyou-7 uniquely expressed DEGs, the top three enriched go terms were “chloroplast thylakoid membrane” (GO:0009535, CC, 25 DEGs), “thylakoid” (GO:0009579, CC, 16 DEGs), and “membrane” (GO:0016020, CC, 54 DEGs) in the S3 stage, “chloroplast” (GO:0009507, CC, 129 DEGs), “aromatic amino acid family biosynthetic process” (GO:0009073, BP, six DEGs), and “chorismate biosynthetic process”(GO:0009423, BP, five DEGs) in the S4 stage, and “serine O-acetyltransferase activity” (GO:0009001, MF, four DEGs), “cellular response to heat” (GO:0034605, BP, 16 DEGs), and “chloride transmembrane transport”(GO:1902476, BP, four DEGs) in the S5 stage. The KEGG pathway analysis (Supplementary Table 7) revealed that the commonly expressed DEGs in Longyou-7 and Tianyou-4, but not in Lenox, were significantly enriched in “carbon fixation in photosynthetic organisms” (brp00710, eight DEGs), “brassinosteroid biosynthesis” (brp00905, three DEGs), and “photosynthesis” (brp00195, six DEGs) pathways in the S3 stage. In the S4 stage, the significantly enriched pathways were “sulfur metabolism” (brp00920, 11 DEGs), “proteasome” (brp03050, 10 DEGs), and “flavonoid biosynthesis” (brp00941, six DEGs). In the S5 stage, the top three enriched pathways were “photosynthesis” (brp00195, 15 DEGs), “phenylalanine, tyrosine, and tryptophan biosynthesis” (brp00400, 13 DEGs), and “photosynthesis-antenna proteins” (brp00196, eight DEGs). For the DEGs uniquely expressed in Longyou-7, the prominently enriched pathways were “fatty acid degradation” (brp00071, seven DEGs), “glycerolipid metabolism” (brp00561, seven DEGs), and “limonene and pinene degradation” (brp00903, two DEGs) in the S3 stage; “photosynthesis-antenna proteins” (brp00196, four DEGs), “proteasome” (brp03050, eight DEGs), and “aminoacyl-tRNA biosynthesis” (brp00970, seven DEGs) in the S4 stage, and “basal transcription factors” (brp03022, 10 DEGs), “spliceosome” (brp03040, 22 DEGs), and “plant-pathogen interaction” (brp04626, 25 DEGs) in the S5 stage. The results suggested that DEGs that participated in photosynthesis-related pathways may confer the stronger winter resistance of the Longyou-7 and Tianyou-4 varieties than that of Lenox, and that the “spliceosome” pathway may contribute to the super winter resistance of Longyou-7.

### Identification of DEGs in Different Stages of the Longyou-7 and Tianyou-4 Versus Lenox

**Figure 5:**
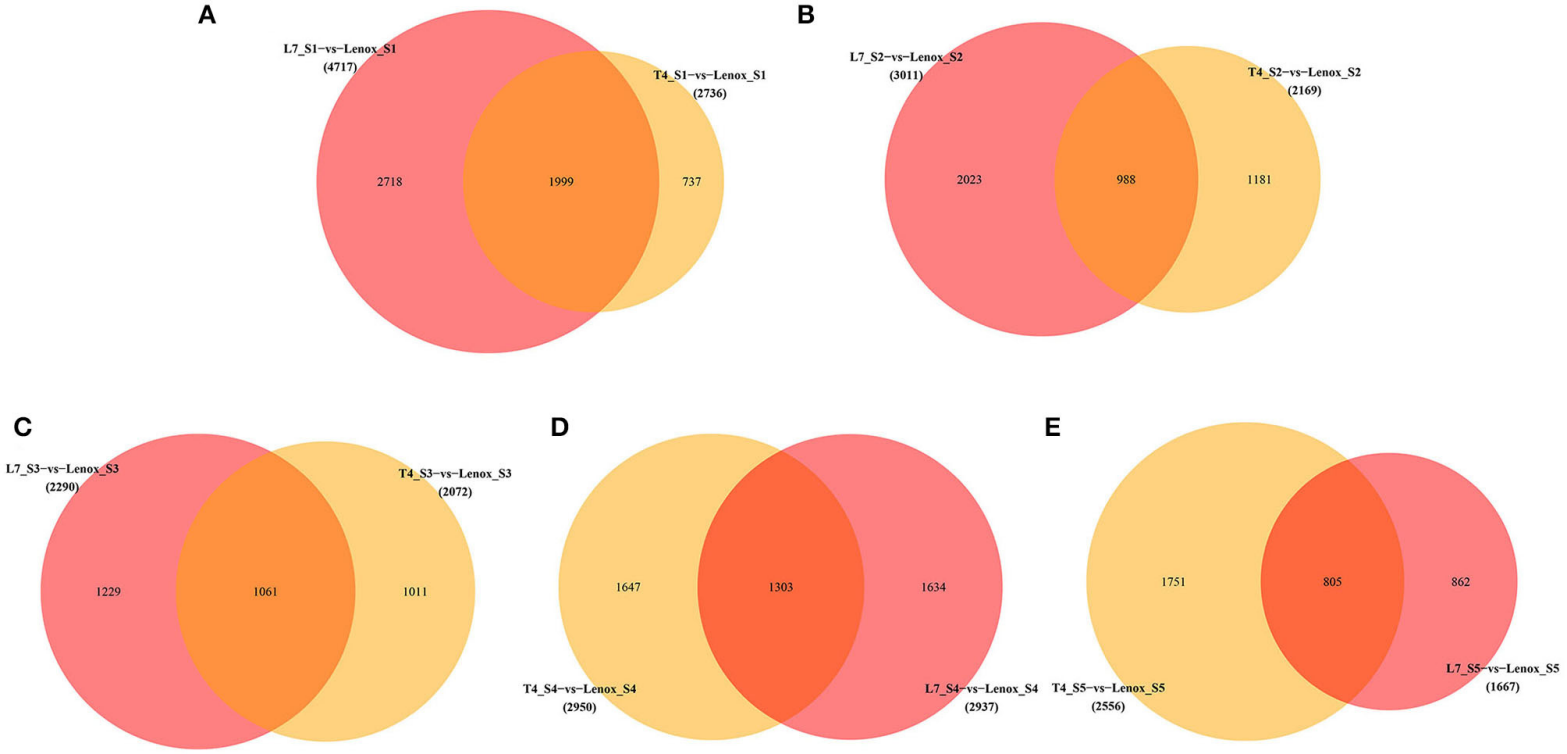
Venn diagrams of differently expressed genes in Longyou-7 (L7) and Tianyou-4 (T4) compared to Lenox in the same stage. **(A)** S1 stage, **(B)** S2 stage, **(C)** S3 stage, **(D)** S4 stage, and **(E)** S5 stage.

Pairwise comparisons were also carried out to identify DEGs in the three varieties in the different sampling (S1–S5) stages (Figure 5). Compared to Lenox, the number of commonly expressed DEGs in the S1 stage was 1,999, with 2,718 and 737 uniquely expressed DEGs in the Longyou-7 and Tianyou-4 varieties, respectively (Figure 5A). In the S2 stage (Figure 5B), 988 DEGs were identified as commonly expressed in Longyou-7 and Tianyou-4, and 2,023 and 1,181 DEGs were specifically expressed in both varieties, respectively. In the S3–S5 stages (Figures 5C–E), compared to Lenox, the number of commonly expressed DEGs in Longyou-7 and Tianyou-4 was 1,061, 1,303, and 805 in the S3, S4, and S5 stages, respectively, with 1,229, 1,634, and 862 DEGs uniquely expressed in Longyou-7 and 1,011, 1,647, and 1,751 DEGs uniquely expressed in Tianyou-4 in the S3–S5 stages.

The KEGG classification analysis showed that DEGs of the commonly expressed genes in Longyou-7 and Tianyou-4 were mostly enriched in carbohydrate metabolism in all the five stages (Supplementary Table 8). The top three enriched pathways of the commonly expressed genes were photosynthesis-antenna proteins (brp00196), photosynthesis (brp00195), and fatty acid elongation (brp00062) in the S1 stage, photosynthesis-antenna proteins (brp00196), aminoacyl-tRNA biosynthesis (brp00970), and plant hormone signal transduction (brp04075) in the S2 stage, starch and sucrose metabolism (brp00500), glyoxylate and dicarboxylate metabolism (brp00630), and circadian rhythm-plant 5 (brp04712) in the S3 stage, tryptophan metabolism (brp00380), photosynthesis-antenna proteins (brp00196), and amino sugar and nucleotide sugar metabolism (brp00520) in the S4 stage, and glucosinolate biosynthesis (brp00966), tryptophan metabolism (brp00380), and starch and sucrose metabolism (brp00500) in the S5 stage (Supplementary Table 9). The KEGG enrichment analysis showed that Longyou-7 and Tianyou-4 also owned unique response pathways to overwinter (Supplementary Table 9). For Longyou-7, the specifically identified DEGs were predominantly enriched in pathways of photosynthesis (brp00195) and photosynthesis-antenna proteins (brp00196) in the S1–S5 stages. Besides that, the specifically identified DEGs in Longyou-7 were also significantly enriched in starch and sucrose metabolism (brp00500) pathway in the S1 stage, phenylpropanoid biosynthesis (brp00940) and pentose and glucuronate interconversions pathways (brp00040) in the S2 stage, glycine, serine, and threonine metabolism pathway (brp00260) in the S3 stage, phenylpropanoid biosynthesis (brp00940) pathway in the S4 stage, and protein processing in the endoplasmic reticulum (brp04141) pathway in the S5 stage. For Tianyou-4, the uniquely identified DEGs were prominently enriched in flavonoid biosynthesis (brp00941), glucosinolate biosynthesis (brp00966), and ubiquinone and other terpenoid-quinone biosynthesis (brp00130) pathways in the S1 stage, glucosinolate biosynthesis (brp00966), flavonoid biosynthesis (brp00941), and cutin, suberine and wax biosynthesis (brp00073) pathways in the S2 stage, glycerophospholipid metabolism (brp00564), sesquiterpenoid and triterpenoid biosynthesis (brp00909), and steroid biosynthesis (brp00100) pathways in the S3 stage, plant hormone signal transduction (brp04075), glycerophospholipid metabolism (brp00564), and phenylpropanoid biosynthesis (brp00940) pathways in the S4 stage, and galactose metabolism (brp00052), starch and sucrose metabolism (brp00500), and glycerophospholipid metabolism (brp00564) pathways in the S5 stage. Thus, the genes in the root collar (including the shoot apical meristem) involved in the photosynthetic and carbohydrate metabolism process may participate in the overwintering memory mechanism of *Brassica rapa* L.

### Expression Pattern of the DEGs in Different Stages of Longyou-7 and Tianyou-4

**Figure 6:**
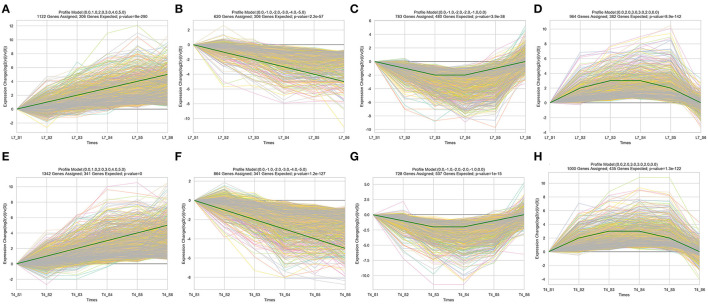
Four representative expression patterns that may be candidate overwintering memory genes were screened in the six sampling stages of **(A–D)** Longyou-7 (L7) and **(E–H)** Tianyou-4 (T4).

**Figure 7:**
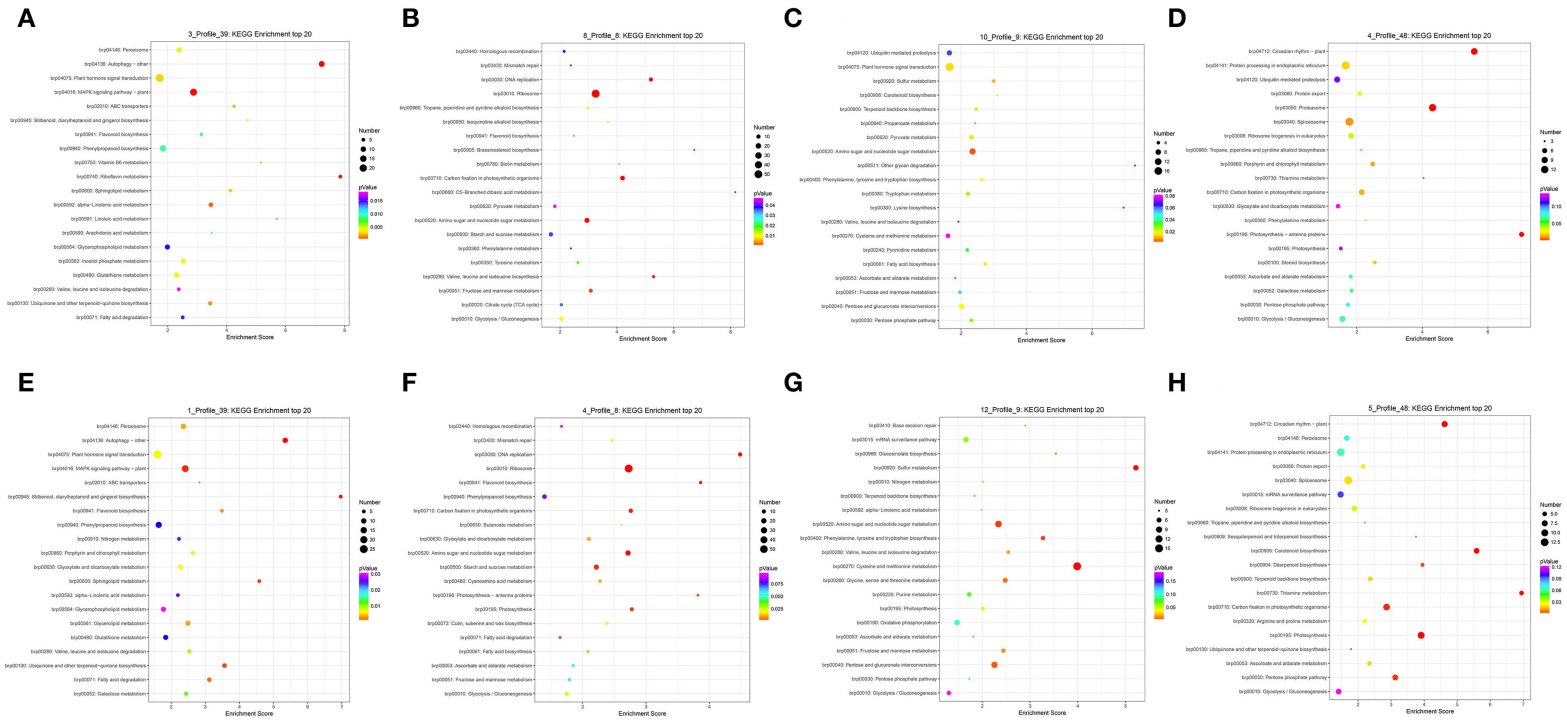
Top 20 of the KEGG pathway enrichment of four representative expression patterns that may be candidate overwintering memory genes in **(A–D)** Longyou-7 (L7) and **(E–H)** Tianyou-4 (T4).

To identify the candidate overwintering memory genes, a STEM analysis was performed to investigate the expression pattern of DEGs in different sampling stages in Longyou-7 and Tianyou-4. According to the expression profiles, the DEGs in the two varieties were classified into 50 groups, and 14 and 12 groups exhibited significant enrichment trends in Longyou-7 and Tianyou-4, respectively (Supplementary Figures 4, 5). For the continuously upregulated expression profiles, 1,122 genes in Longyou-7 and 1,342 genes in Tianyou-4 were classified into profile 39 (Figures 6A,E), and we defined this expression pattern as “+, +” candidate memory genes. The KEGG pathway results showed that most of the genes in both Longyou-7 and Tianyou-4 participated in the “plant hormone signal transduction” (brp04075), “MAPK signaling pathway-plant” (brp04016), and “phenylpropanoid biosynthesis” (brp00940) pathways (Figures 7A,E, Supplementary Table 10). For the persistently downregulated expression profiles (defined as “–, –” candidate memory genes), Longyou-7 and Tianyou-4 contained 620 and 864 genes, respectively, that were classified into profile 8 (Figures 6B,F). The majority of the genes in this expression pattern was significantly enriched in the “ribosome” (brp03010) and “amino sugar and nucleotide sugar metabolism” (brp00520) pathways in both varieties (Figures 7B,F, Supplementary Table 10). There were 783 genes in Longyou-7 and 728 genes in Tianyou-4 classified into profile 9, and the expression level of these genes continued to decrease until the S3 stage and then increased, and they were defined as “–, +” candidate memory genes (Figures 6C,G). The genes in Longyou-7 were mainly involved in the “plant hormone signal transduction” (brp04075), “amino sugar and nucleotide sugar metabolism” (brp00520), and “pentose and glucuronate interconversions” (brp00040) pathways, whereas the genes in Tianyou-4 were mostly enriched in the “cysteine and methionine metabolism” (brp00270), “pentose and glucuronate interconversions” (brp00040), and “amino sugar and nucleotide sugar metabolism” (brp00520) pathways. The “plant hormone signal transduction” pathway was only significantly enriched in Longyou-7 (Figures 7C,G, Supplementary Table 10). We also found 964 genes in Longyou-7 and 1,000 genes in Tianyou-4 classified into profile 48 whose expression level continued to rise until the S3 stage and then fell, and they were defined as “+, –” candidate memory genes (Figures 6D,H). The majority of the genes in Longyou-7 was significantly enriched in the “spliceosome” (brp03040), “protein processing in endoplasmic reticulum” (brp04141), “proteasome” (brp03050), and “circadian rhythm-plant” (brp04712) pathways; however, in Tianyou-4, the genes were significantly enriched in the “spliceosome” (brp03040), “protein processing in endoplasmic reticulum” (brp04141), “photosynthesis” (brp00195), “carbon fixation in photosynthetic organisms” (brp00710), and “circadian rhythm-plant” (brp04712) pathways (Figures 7D,H, Supplementary Table 10). The four distinct transcriptional expression patterns of candidate overwintering memory genes imply that stress memory may be accomplished in the S3 stage and store information on previous stresses to cope with the subsequent more severe winter cold exposure. All the above information reveals the pattern of candidate memory gene expression and lays some foundation for further confirmation of the overwintering memory genes and detailed analysis of molecular mechanisms regulating the root development and winter memory of *Brassica rapa* L.

### Changes in Transcription Factors and Genetic Variations in Different Stages of the Three Varieties

As critical regulators that coordinate the expression of genes involved in plant development and various abiotic stress responses, transcription factors (TFs) were also identified in this study (Supplementary Table 11). The results showed that 58 TF families existed in the three varieties, and that the *WRKY* family made up the majority in all TF groups in different stages. Besides the *WRKY* family, the other top 12 differentially expressed transcription factor families were the *bHLH, C2H2, MYB, ERF, B3, MYB-related, C3H, NAC, G2-like, bZIP*, and *HD-ZIP* families (Supplementary Figure 6). In the *WRKY, MYB, C3H, NAC*, and *bZIP* families, more TFs were upregulated than downregulated, while in the *bHLH, C2H2, ERF, B3, MYB-related, G2-like*, and *HD-ZIP* families, downregulated TFs were dominant. These TFs families, especially the *WRKY* family, may play an important role in regulating genes involved in the overwintering process.

Furthermore, genetic variations in SNPs/INDELs that may affect the root development and overwintering ability of the three varieties were also analyzed. A total of 2,318,336/263,358, 2,449,729/276,546, and 2,053,925/229,239 SNPs/INDELs were identified in Longyou-7, Tianyou-4, and Lenox, respectively. Venn diagrams were established to identify specific SNPs/INDELs of the three varieties (Supplementary Figure 7). The Results showed that 506,477/91,364, 585,080/100,095, and 526,641/93,174 unique SNPs/INDELs existed in Longyou-7, Tianyou-4, and Lenox, respectively. In each sample, there was an average of 751,731 (428,933 transitions and 322,798 transversions), 794,981 (455,130 transitions and 339,852 transversions), and 766,883 (441,587 transitions and 325,296 transversions) SNPs identified in Longyou-7, Tianyou-4, and Lenox, respectively, and transition events occurred more frequently than transversion events in all the three varieties (Supplementary Table 12). The average number of INDELs in each sample of Longyou-7, Tianyou-4, and Lenox was 51,066, 51,489, and 49,242. The SNPs were mainly distributed in exon regions, whereas the INDELs were mostly located in UTRs and upstream regions (Supplementary Table 12). In addition, an analysis of unique SNPs/INDELs in the three varieties in different sampling stages was executed. The number of unique SNPs/IINDELs in Longyou-7 with strong overwintering ability was less than that in Tianyou-4 and Lenox (Supplementary Table 12). Furthermore, to test whether the SNP/INDELs were associated with DEGs, SNPs/INDELS with significant difference in pairwise comparisons of the three varieties and different stages were also detected, and gene names of the different SNPs/INDELS were acquired by gene annotation. The KEGG enrichment analysis showed that most of the intersections between the candidate genes for different SNPs/INDELS and DEGs were also significantly involved in the plant hormone signal transduction, starch and sucrose metabolism, photosynthesis, carbon fixation in photosynthetic organisms, amino sugar and nucleotide sugar metabolism, phenylpropanoid biosynthesis, MAPK signaling pathway-plant, and ribosome pathways (Supplementary Table 12). Moreover, the ASprofile software was used to survey for alternative splicing events (Supplementary Table 13). There were 838,608, 909,311, and 749,833 alternative splicing (AS) events in Longyou-7, Tianyou-4, and Lenox, respectively. A total of 103,765 shared AS events were jointly owned by the three varieties. Furthermore, 550,918, 605,706, and 492,585 AS events were identified only in Longyou-7, Tianyou-4, and Lenox, respectively (Supplementary Figure 7). Moreover, the average frequency of AS events in each sample was 75,142, 74,090, and 71,513 in Longyou-7, Tianyou-4, and Lenox, respectively, and the major AS events were due to alternative last exons (TTS), alternative first exons (TSS), intron retention (IR), and alternative exon ends (AE) (Supplementary Table 13). The above results indicated that the spliceosome pathway might contribute to the super winter resistance of Longyou-7, and then Longyou-7-specific AS events were analyzed in different stages (Supplementary Table 13), which are genetic basis for identification of winter cold-specific AS events. Simultaneously, the rMATS method was used to detect the differential AS events in Longyou-7 in different stages compared to the S1 stage. The KEGG enrichment analysis of candidate genes with different AS events indicated that the candidate genes were mainly involved in the spliceosome pathway (Supplementary Table 13). The AS events that occurred in these candidate genes related to the spliceosome pathway in Longyou-7 might be winter cold-specific. Taken together, the identified SNPs/INDELs and alternative splicing events will provide valuable genetic resources for future winter cold-specific trait analysis of *Brassica rapa* L.

### Validation of Candidate Overwintering Memory Genes by qRT-PCR Analysis

To validate the expression patterns of memory genes and the reliability of transcriptome data, 11 genes belonging to the four different expression patterns of candidate overwintering memory genes were selected for qRT-PCR, including five “+, +” candidate memory genes that belonged to plant hormone signal transduction pathway (*LOC103833785, LOC103874395, LOC103860324, LOC103845633*) and phenylpropanoid biosynthesis (*LOC103865042*), three “–, –”candidate memory genes associated with amigo sugar and nucleotide sugar metabolism (GME, *LOC103846395*) and starch and sucrose metabolism (*LOC103831107*), one “–, +” candidate memory genes involved in pentose and glucuronate interconversions pathway, and two “+, –” candidate memory genes associated with photosynthesis (*LOC103846718*) and spliceosome (*LOC103847147*) pathways. The results showed that the qRT-PCR expression results were generally consistent with RNA-Seq data with a good positive correlation (*R*^2^ = 0.88014) (Supplementary Figure 8), which confirmed the reliability of the transcriptome data in the present study.

## Discussion

Winter rapeseed can now grow in the cold and arid regions of northwest China with breakthroughs in the breeding of cold-resistant varieties, and is the only overwintering cruciferous oilseed crop in the north area of China to bring significant economic and ecological benefits (Sun et al., 2007a, 2010). Winter turnip rape (*Brassica rapa* L.) is generally sown in the middle of August in the northwest region of China. It requires undergoing a long overwintering period to turn green in the spring of the next year. Roots, including the shoot apical meristem, as the only living organ during prolonged cold exposure in winter, are critical for the overwintering ability and reconstruction of the above-ground organ once it perceives warm temperatures in spring (Liu et al., 2013). Although previous studies have investigated the morphological structures and physiological, biochemical, and molecular mechanisms (Yang et al., 2014; Zeng et al., 2018a,b; Ma et al., 2019; Xu et al., 2020b; Niu et al., 2021) of cold stress in *Brassica rapa* L., little is known about its intricate mechanism of overwintering memory. Therefore, in the present study, the overwintering memory mechanisms were investigated by the analysis of root collar tissues (including the shoot apical meristem) of Longyou-7 (strong cold tolerance), Tianyou-4 (middle cold tolerance), and Lenox (cold-sensitive) by transcriptome. This study will provide an insight into the complex overwintering memory mechanisms of winter rapeseed and important genetic resources for the molecular breeding of cold-resistant varieties.

### Winter Rapeseed Root Development Is Largely Determined in an Early Overwintering Period

It is well-known that roots play an important role in determining the safe overwintering ability of winter rapeseed (Sun et al., 2007b; Wu et al., 2014). In the present study, we analyzed the root collar diameter of three rapeseed varieties, Longyou-7, Tianyou-4, and Lenox, with contrasting overwintering abilities over a time course of prolonged winter cold exposure (S1–S5 stages). The root collar diameter of Longyou-7 was larger than that of Tianyou-4, but Tianyou-4 was larger than Lenox. Furthermore, in the S2 stage, root development was significantly increased compared to that in the S1 stage, but the roots developed slowly in the S3–S5 stages (Figure 1). These results suggested that root development differs between different winter rapeseed varieties. The cold-resistant varieties may preferentially assign more photosynthetic products to the underground parts to store more organic matters for completing root development (Sun et al., 2007b) and limit the growth of aboveground leaves in the early overwintering period, thus improving its overwintering ability. However, the exact factor determining root development cannot be determined in this study. Further studies on assimilate transport from aboveground to underground parts before winter will help us discover the important factor that defines winter survival.

### Phenylpropanoid Biosynthesis and Plant Hormone Signal Transduction Pathways Likely Contribute to the Difference in Winter Rapeseed Root Development and Overwintering Memory

Molecular mechanisms underlying overwintering memory are poorly understood in winter rapeseed. We conducted RNA-seq analyses on root collars (including the shoot apical meristem) to detect the global transcriptome dynamics in three rapeseed varieties (with contrasting overwintering ability) in different winter cold exposure and regreening stages, and investigated the molecular mechanism underlying the difference in root development and overwintering memory. The transcriptome data implied significant changes at different stages of the three varieties, and the difference at the S2 and S3 stages may closely relate to root development and overwintering abilities by principal component analysis (PCA) and hierarchical clustering analysis (Figure 2). Principal component 1 clearly separated the samples from different stages, and principal component 2 separated Lenox from Longyou-7 and Tianyou-4. The KEGG enrichment analysis showed that most DEGs involved in the phenylpropanoid biosynthesis (brp00940) and plant hormone signal transduction (brp04075) pathways had significant differences in the S2 stage of the three varieties (Supplementary Table 6).

Phenylpropanoid biosynthesis is the critical metabolic pathway for synthesizing secondary metabolites, which are closely related to the development of and stress response in plants (Baxter and Stewart, 2013). In many instances, plants adjust their growth and response to abiotic and biotic stresses by altering the expression of genes involved in the phenylpropanoid biosynthesis pathway (Li et al., 2020; Gan et al., 2021; Meng et al., 2021; Zhu Y. et al., 2021). Previous studies found that cold stress influenced the expression of genes involved in the phenylpropanoid biosynthesis pathway of winter rapeseed (Zeng et al., 2018b; Ma et al., 2019). In the present study, many DEGs were enriched in “phenylpropanoid biosynthesis (brp00940)” in the S2 stage compared to the S1 stage, especially the branch pathway synthesizing the secondary metabolites of lignins. In Longyou-7, the phenylpropanoid biosynthesis pathway appeared in the top 20 of both the upregulated and downregulated KEGG pathways. In contrast, in Tianyou-4 and Lenox, the phenylpropanoid biosynthesis pathway was only found in the top 20 upregulated KEGG pathways (Supplementary Table 6). It has been reported that a large amount of lignin biosynthesis genes are correlated with plant development (Yoon et al., 2015; Wang et al., 2016) and stress adaptation (Chun et al., 2019). In our study, focusing on expression changes in genes related to lignin biosynthesis, we found that genes encoding the phenylalanine ammonia lyase (PAL) that initiates lignin biosynthesis by deamination of phenylalanine into cinnamic acid (Boerjan et al., 2003) were upregulated with greater changes in the middle cold tolerance variety Tianyou-4 (*LOC103841251* and *LOC103867229*) and the cold-sensitive variety Lenox (*LOC103863433, LOC103841251*, and *LOC103867229*) than in the cold-resistant variety Longyou-7 with no significant change. Additionally, genes related to hydroxycinnamoyl-CoA shikimate/quinate hydroxycinnamoyl transferase (HCT, *LOC103856729*) and ferulate 5-hydroxylase (F5H, *LOC103869607*) were only upregulated in Tianyou-4, and are the key enzymes for synthesis of H-lignin (Vanholme et al., 2010) and S-lignin (Stewart et al., 2009), respectively. Moreover, DEGs encoding 4-coumarate-CoA ligase (4CL) were downregulated in Longyou-7 (*LOC103869196*) and upregulated in Tianyou-4 (*LOC103869197*) and Lenox (*LOC103869196*) (Supplementary Table 14). Importantly, of the 11 specifically expressed DEGs in Longyou-7 in the S2 stage (Supplementary Table 7), four were associated with cinnamyl alcohol dehydrogenase (CAD: *LOC103828799, LOC103835065, LOC103840386*, and *LOC103867418*) and peroxidase (PER/LAC: *LOC103837896, LOC103869716, LOC103846031*, and *LOC103847421*). In Longyou-7, more genes related to peroxidase (PER/LAC) were downregulated; however, in Tianyou-4 and Lenox, the DEGs related to peroxidase (PER/LAC) were upregulated. It has been reported that reduced expression level of genes related to lignin biosynthesis may lead to decreased lignin content to satisfy root growth (Wang et al., 2016). Taken together, we inferred that the DEGs involved in lignin biosynthesis may play significant roles in the root development of winter rapeseed. However, the regulatory mechanisms of the phenylpropanoid biosynthesis pathway, especially lignin biosynthesis branches in the root development and overwintering ability of winter rapeseed, need to be demonstrated by further studies.

In general, phytohormones, including ethylene (ETH), auxin (AUX), abscisic acid (ABA), gibberellin (GA), cytokinin (CK), brassinosteroid (BR), and jasmonic acid (JA), are central regulators and interact with each other during root growth and development in plants (Lewis et al., 2011; Liu et al., 2017; Qin et al., 2019; Xu P. et al., 2020). Similar results were obtained in the present study. A number of DEGs were enriched in “plant hormone signal transduction (brp04075)” in the S2 stage compared to the S1 stage. In Longyou-7 and Tianyou-4, the plant hormone signal transduction pathway only appeared in the top 20 downregulated KEGG pathways. In contrast, the plant hormone signal transduction pathway was found in the top 20 of both the upregulated and downregulated KEGG pathways in Lenox (Supplementary Table 6). A total of 34 and 32 downregulated DEGs were enriched in the plant hormone signal transduction pathway in Longyou-7 and Tianyou-4, respectively. In Lenox, there were 28 upregulated DEGs and 35 downregulated DEGs enriched in the plant hormone signal transduction pathway (Supplementary Table 6). Among them, 20 and 12 downregulated DEGs, and 10 upregulated and 19 downregulated DEGs encoding “auxin transporter protein 1,” “AUX/IAA family,” “auxin responsive GH3 gene family,” and “auxin-responsive protein SAUR family” that belonged to auxin signaling were found in Longyou-7, Tianyou-4, and Lenox, respectively. Furthermore, nine of the 20 specifically expressed DEGs (AUX/IAA: *LOC103834036, LOC103868947, LOC103864374, LOC103849138, LOC103843664*; SAUR: *LOC103856282, LOC103860467, LOC103834602*, and *LOC103859470*) in Longyou-7 in the S2 stage (Supplementary Table 7) were downregulated and participated in auxin signaling. Therefore, we speculate that auxin might be closely related to the root development of winter rapeseed. It is known that auxin is a core regulator integrating other plant hormones in root development (Xu P. et al., 2020). Previous studies have shown that auxin can interact synergistically with ethylene to inhibit primary root growth (Strader et al., 2010; Hu et al., 2017; Qin and Huang, 2018). RuŽicka et al. (2007) found that ethylene accelerates the accumulation of auxin to inhibit the root development of *Arabidopsis* by stimulating the expression of auxin biosynthesis and transport-related genes. Interestingly, we found that in the cold-tolerant varieties, two DEGs encoding ethylene receptor ETHYLENE RESISTANT1 (ETR, *LOC103833878*) and master transcriptional regulator ETHYLENE INSENSITIVE 3 (EIN3, *LOC103858337*) in Longyou-7 and one ETR DEG (*LOC103833878*) in Tianyou-4 were downregulated. However, in the cold-sensitive variety Lenox, the transcriptional regulator EIN3 (*LOC103855853*) was upregulated, resulting in high expression of one ethylene response factor (ERF1/2, *LOC103833785*). Moreover, the mitogen-activated protein kinase (SIMKK, *LOC103859896*) was downregulated, and EIN3-BINDING F BOX PROTEIN 1/2 (EBF1/2, *LOC103837087*) was upregulated. Mao et al. (2016) found that ERF1 can directly activate ANTHRA NILATE SYNTHASE α1 (ASA1) to increase the biosynthesis of auxin and suppress root growth in *Arabidopsis*; thus, ERF1 may act as a pivotal junction connecting the ethylene and auxin signal to inhibit the root development (Xu P. et al., 2020) of the cold-sensitive variety Lenox. It is known that ABA participates in plant growth and development regulation in many ways (Qin et al., 2019), and it has dual roles in root growth depending on ABA concentration by interacting with the ethylene and auxin signaling pathways (Li et al., 2017). In the middle cold-tolerant variety Tianyou-4, we found that four core components of the ABA signaling pathway were all upregulated, namely, three PYRABACTIN RESISTANCE1 (PYR1)/PYR1-LIKE (PYL) DEGs (*LOC103844644, LOC103827766*, and *LOC103858828*), two protein phosphatase 2C (PP2C) DEGs (*LOC103834849* and *LOC103845336*), four protein kinases SnRK2 DEGs (*LOC103843292, LOC103854644, LOC103873775*, and *LOC103864444*), and one ABRE-BINDING FACTOR (ABF, LOC103867300). In the strong cold-tolerant variety Longyou-7, we identified two PYR/PYL DEGs (downregulated: *LOC103857100*, upregulated: *LOC103827766*), two PP2C DEGs (downregulated: *LOC103843946*, upregulated: *LOC103845336*), two upregulated SnRK2 DEGs (*LOC103854644* and *LOC103864444*), and one upregulated ABF DEG (*LOC103865861*). Meanwhile, we also discovered three identical upregulated PYR/PYL DEGs in Tianyou-4, two PP2C DEGs (downregulated: *LOC103870352*, up-regulated: *LOC103870352*), two upregulated SnRK2 DEGs in Longyou-7, and one downregulated ABF DEG (*LOC103844297*) in the cold-sensitive variety Lenox (Supplementary Table 14). Furthermore, DEGs involved in the BR, CK, GA, JA, and SA pathways were also identified in the present study (Supplementary Table 14). These results are in accordance with what has been found in our previous research studies, which suggested that contents of ABA and IAA play important roles in cold tolerance of *Brassica rapa* L. grown in the field (Xu et al., 2020b; Niu et al., 2021). Therefore, we speculate that phytohormones, especially the auxin, ethylene, and ABA signaling pathways, can coordinate to regulate the root development and overwintering ability of winter rapeseed.

Moreover, in our study, most of the continuously upregulated (“+, +”) candidate overwintering memory genes enriched in the plant hormone signal transduction, MAPK signaling pathway-plant, and phenylpropanoid biosynthesis pathways were identified in both Longyou-7 and Tianyou-4 (Figures 7A,E, Supplementary Table 10). Our data indicated that the phenylpropanoid biosynthesis and plant hormone signal transduction pathways likely contribute to the difference in root development and overwintering memory of *Brassica rapa* L.

### Photosynthesis and Carbohydrate Metabolism Processes Also Potentially Contributed to the Overwintering Memory of *Brassica rapa* L.

In general, because of daily and seasonal fluctuations in photoperiods and temperatures under natural conditions (Li et al., 2021; Chen et al., 2022), winter annual plants have generated intricate mechanisms to adjust their growth and development in response to climate changes. A changed photoperiod has an important regulating role in photosynthesis activity (Bauerle et al., 2012). Photosynthesis is a process in which plants use sunlight to convert CO_2_ into soluble carbohydrates to acquire energy for subsequent plant growth and development (Simkin et al., 2020), and it is also involved in low temperature response (Sharma et al., 2020). Previous studies have also reported that photosynthesis and circadian rhythm pathways are enriched in field cold acclimation in winter of overwintering evergreen (Liu et al., 2020), and PSII reaction centers are closed/degraded in winter with the gradual cooling occurrence in overwintering plants (Liu et al., 2020; Chang et al., 2021). In addition, we also found that the photosynthetic performance of winter rapeseed declined under winter cold exposure (Xu et al., 2020a). In line with the above-mentioned studies, it was shown that photosynthesis, carbon fixation in photosynthetic organisms, and photosynthesis-antenna proteins were the key pathways enriched by commonly expressed DEGs in the Longyou-7 and Tianyou-4 varieties but not in Lenox in the S3–S5 stages compared to the S1 stage (Supplementary Table 7). As chloroplast is the best-known site for photosynthesis (Cackett et al., 2021), our transcriptome data also showed that the commonly expressed DEGs in Longyou-7 and Tianyou-4 in the S3–S5 stages compared to the S1 stage were associated with chloroplast thylakoid, chloroplast thylakoid membrane, and chloroplast by the GO analysis (Supplementary Table 7). Moreover, the “+, –” candidate memory genes were also found enriched in the circadian rhythm-plant, photosynthesis, and carbon fixation in photosynthetic organism pathways in both Longyou-7 and Tianyou-4 (Figure 7H, Supplementary Table 10). In Longyou-7 and Tianyou-4, 10 and eight candidate genes, respectively, were found to be participating in the circadian rhythm-plant pathway, encoding the two-component response regulator-like APRR5 (Longyou-7/Tianyou-4: *LOC103854859* and *LOC103874318*), two-component response regulator-like APRR1 (Longyou-7/Tianyou-4: *LOC103860589* and *LOC103837378*), two-component response regulator-like APRR9 (Longyou-7: *LOC103866306*; Tianyou-4: *LOC103866432*), protein EARLY FLOWERING 3 (Longyou-7: *LOC103864705* and *LOC103249168*; Tianyou-4: *LOC103864705*), transcription factor HY5 (Longyou-7/Tianyou-4: *LOC103846737*), protein suppressor of PHYA-105 1 (SPA1, Longyou-7: *LOC103866477*), phytochrome A (Longyou-7: *LOC103871728*), and cryptochrome-1 isoform X2 (Tianyou-4: *LOC103844260*). Among the candidate genes involved in the photosynthesis pathway, there are two photosystem II-related proteins (PsbY: *LOC103831199*; PsbS: *LOC103847722*), one photosystem I-related protein (PsaG: *LOC103832566*), and one cytochrome b6-f complex subunit (PetC: *LOC103846718*) in Longyou-7, and five photosystem II-related proteins (PsbR: *LOC103853163*; PsbW: *LOC103867946*; PsbY: *LOC103831199*; PsbS: *LOC103847722*; PsbO: *LOC103855346*), two photosynthetic electron transport subunits (PetJ: *LOC103848470*; PetF: *LOC103868066, LOC103840153*), and one cytochrome b6-f complex subunit (PetC: *LOC103858708, LOC103846718*) in Tianyou-4. For the carbon fixation in photosynthetic organism pathway, candidate genes mainly encoded the ribulose bisphosphate carboxylase small chain (Longyou-7: *LOC103863779, LOC117133689*, and *LOC103863782*; Tianyou-4: *LOC103863779, LOC103863782*, and *LOC103864160*), fructose-1,6-bisphosphatase (Longyou-7: *LOC103863263* and *LOC103841316*; Tianyou-4: *LOC103841316*), malate dehydrogenase (Longyou-7: *LOC103851645*; Tianyou-4: *LOC103851645* and *LOC103856701*), glyceraldehyde-3-phosphate dehydrogenase (Longyou-7: *LOC103843097*; Tianyou-4: *LOC103875281* and *LOC103843097*), and fructose-bisphosphate aldolase 1 (Tianyou-4: *LOC103864267*).

Cackett et al. (2021) also indicated that phytohormones are coordinators of chloroplast development throughout plant growth and can affect chloroplast- and photosynthesis-related gene expression. It has been reported that the ABA signal transduction pathway plays a key role in freezing stress during overwintering of *Camellia sinensis* (Wu et al., 2021). In this study, we also found that genes related to the plant hormone signal transduction pathway were enriched by all the four types of candidate overwintering memory genes in both Longyou-7 and Tianyou-4 (Supplementary Table 10), thus implying that the plant hormone signal transduction pathways might interact with the photosynthesis process to regulate overwintering memory. In this study, candidate memory genes involved in the plant hormone signal transduction pathway were assigned to the abscisic acid (ABA), auxin (AUX), and ethylene (ETH) signal transduction pathways. For the ABA signal transduction pathway, nine (PP2C: *LOC103863846, LOC103861611*, and *LOC103834849*; PYR/PYL: *LOC103867695, LOC103855626*, and *LOC103830315*; SnRK2: *LOC103871868* and *LOC103829556*; ABF: *LOC103859804*) and six (PP2C: *LOC103834849*; PYR/PYL: *LOC103867695* and *LOC103830315*; SnRK2: *LOC103871868*; ABF: *LOC103865861* and *LOC103869470*) genes belonged to the “+, +” expression pattern, and three (SnRK2: *LOC103864444* and *LOC103847173*; PYR/PYL: *LOC103827766*) and four (PYR/PYL: *LOC103864102* and *LOC103827766*; PP2C: *LOC103845336*; ABF: *LOC103841580*) genes belonged to the “+, –” expression pattern in Longyou-7 and Tianyou-4, respectively. Among the candidate genes involved in the ETH signal transduction pathway, six and two genes belonged to the “+, +” expression pattern and the “–, +” expression pattern in Longyou-7, respectively, encoding ethylene-responsive transcription factor (ERF1/2, “+, +” pattern: *LOC103833785, LOC103844067*, and *LOC103827660*), EIN3-binding F-box protein (EBF1/2, “+, +” pattern: *LOC103864667* and *LOC103874395*), ethylene response sensor 2 (ETR, “+, +” pattern: *LOC103836581*; “–, +” pattern: *LOC103844270*), and ETHYLENE INSENSITIVE 3-like 1 protein (EIN3, “–, +” pattern: *LOC103858337*). For the candidate genes of the ETH signal pathway in Tianyou-4, there were only four genes (ERF1/2: *LOC103833785*; EIN3: *LOC103859874*; EBF1/2: *LOC103874395*; ETR: *LOC103831098*) belonging to the “+, +” expression pattern. Additionally, the AUX signal transcription pathway was enriched by all the four types of candidate genes. For the “+, +” expression profile, Longyou-7 and Tianyou-4 contained one (GH3: *LOC103866974*) and three (GH3: *LOC103866974*; SAUR: *LOC103862867, LOC103872443*) genes, respectively. For the “–, –” expression profile, two (SAUR: *LOC103862456*; AUX/IAA: *LOC103836586*) and eight (SAUR: *LOC103862373* and *LOC103834917*; GH3: *LOC103866348, LOC103838986*, and *LOC103871837*; ARF: *ARF18-2* and *ARF18-1*; AUX/IAA: *LOC103871247*) genes belonged to the AUX pathway in Longyou-7 and Tianyou-4, respectively. Moreover, there were eight genes (AUX/IAA: *LOC103854273 LOC103840684*, and *LOC103864374*; AUX1: *LOC103857897*; SAUR: *LOC103860183* and *LOC103834917*; ARF: *LOC103829217*; TIR1: *LOC103858715*) in Longyou-7 and eight genes (SAUR: *LOC103860183, LOC103859470, LOC103844834, LOC103846762*, and *LOC103832008*; GH3: *LOC103848751*; ARF: *LOC103829217*; TIR1: *LOC103858715*) in Tianyou-4 classified in the “–, +” expression profile. We also found one gene (AUX/IAA: *LOC103832822*) in Longyou-7 and two genes (AUX/IAA: *LOC103832490* and *LOC103832822*) in Tianyou-4 classified into the “+, –” expression profile.

Moreover, we also identified DEGs in the three varieties in the different sampling (S1–S5) stages and found that DEGs of the commonly expressed genes in Longyou-7 and Tianyou-4 compared to Lenox were mostly enriched in carbohydrate metabolism, especially “starch and sucrose metabolism” and “amino sugar and nucleotide sugar metabolism” as well as photosynthesis-related pathways in all the five stages (Tables S8 and S9). Carbohydrate metabolism has been reported to play a significant role in many plants' physiological processes and stress responses, as it is directly linked to photosynthesis (Xalxo et al., 2020). Similarly, Liu et al. (2020) indicated that photosynthesis, through direct influence on carbohydrate metabolism, mediate the increase of freezing tolerance in overwintering evergreens. Recently, Olas et al. (2021) reported that primary carbohydrate metabolism genes played a crucial role in the heat-stress memory of *Arabidopsis thaliana*, and that thermopriming gave rise to complex changes of metabolic adjustments in starch and sugar metabolism. Consistent with these observations, our results also showed that the persistently downregulated candidate overwintering memory genes (“–, –”) were mostly enriched in the amino sugar and nucleotide sugar metabolism pathway of carbohydrate metabolism and carbon fixation in the photosynthetic organism pathway in both Longyou-7 and Tianyou-4 (Figures 7B,F, Supplementary Table 10). In Longyou-7 and Tianyou-4, 16 and 19 candidate genes, respectively, were found to be involved in the amino sugar and nucleotide sugar metabolism pathways. Among them, many genes encoded the galacturonosyltransferase (Longyou-7: *LOC103843002, LOC103860270*, and *LOC103827612*; Tianyou-4: *LOC103843002* and *LOC103827612*), UDP-arabinopyranose mutase (Longyou-7: *LOC103842932, LOC103856155*, and *LOC103846395*; Tianyou-4: *LOC103842932, LOC103856155, LOC103870925*, and *LOC103846395*), glucose-1-phosphate adenylyltransferase (Longyou-7: *LOC103864283* and *LOC103828545*; Tianyou-4: *LOC103828545*), and GDP-mannose 3,5-epimerase (Longyou-7: *GME*; Tianyou-4: *GME, LOC103854620*). The candidate genes in the carbon fixation in photosynthetic organism pathway were involved in encoding malate dehydrogenase 1 (Longyou-7: *LOC103871139, LOC103832683, LOC103839246*, and *LOC103844258*; Tianyou-4: *LOC103871139, LOC103832683*, and *LOC103839246*), phosphoglycerate kinase (Longyou-7: *LOC103859466, LOC103870225*, and *LOC103832426*; Tianyou-4: *LOC103859466* and *LOC103870225*), fructose-bisphosphate aldolase (Longyou-7: *LOC103841225* and *LOC103842537*; Tianyou-4: *LOC103841225*), glyceraldehyde-3-phosphate dehydrogenase (Longyou-7: *LOC103850055*; Tianyou-4: *LOC103850055* and *LOC103848938*), triosephosphate isomerase (Longyou-7: *LOC103863161*; Tianyou-4: *LOC10386425*2), and phosphoribulokinase (*LOC103868914*). Furthermore, the “–, +” candidate memory genes were mainly enriched in the amino sugar and nucleotide sugar metabolism and pentose and glucuronate interconversion pathways in both Longyou-7 and Tianyou-4 (Figures 7C,G, Supplementary Table 10). In Longyou-7, 10 genes encoding GDP-mannose 3,5-epimerase (*LOC103854620*), endochitinase (*LOC103857315* and *LOC103838693*), UDP-glucose 6-dehydrogenase 4 (*LOC103863829*), mannose-1-phosphate guanyltransferase (*LOC103864220* and *LOC103865745*), mannose-6-phosphate isomerase 1 (*LOC10387090*7), beta-hexosaminidase 3 (*LOC103831061*), UDP-N-acetylglucosamine diphosphorylase 1 (*LOC103833836*), and cellulose synthase-like protein D5 (*LOC103844566*) were enriched in the amino sugar and nucleotide sugar metabolism pathway, and eight genes encoding the probable pectate lyase (*LOC103863605, LOC103850282, LOC103843635*, and *LOC103844229*), pectinesterase/pectinesterase inhibitor 3 (*LOC103842134, LOC103870041*, and *LOC103848519*), and UDP-glucose 6-dehydrogenase 4 (*LOC103863829*) were enriched in the pentose and glucuronate interconversion pathway. In Tianyou-4, 10 candidate genes were involved in the amino sugar and nucleotide sugar metabolism pathway, encoding UDP-glucose 6-dehydrogenase (*LOC103863829* and *LOC103846408*), mannose-1-phosphate guanyltransferase (*LOC103864220* and *LOC103865745*), hexokinase (*LOC103837987* and *LOC103843922*), phosphomannomutase (*LOC103866239*), galacturonosyltransferase 8 (*LOC103828576*), UDP-N-acetylglucosamine diphosphorylase 1 (*LOC103833836*), and basic endochitinase (*LOC10384776*6), whereas nine genes were engaged in the pentose and glucuronate interconversions pathway, encoding the probable pectate lyase (*LOC103863605, LOC103831201, LOC103837270*, and *LOC103843635*), UDP-glucose 6-dehydrogenase (*LOC103863829* and *LOC103846408*), and pectinesterase/pectinesterase inhibitor (*LOC103870041, LOC103870804*, and *LOC103843236*). Besides, the starch and sucrose metabolism pathway was also found to be enriched by all the four expression patterns of overwintering candidate memory genes in both Longyou-7 and Tianyou-4 (Supplementary Table 10), especially by the “–, –” expression pattern, encoding beta-glucosidase (Longyou-7: *LOC103864958* and *LOC103866139*; Tianyou-4: *LOC103838824, LOC103851390, LOC103861251, LOC103864958, LOC10386679*8, and *LOC103847250*), endoglucanase 21 (Longyou-7: *LOC103861904*; Tianyou-4: *LOC103861904* and *LOC103861475*), glucose-1-phosphate adenylyltransferase (Longyou-7: *LOC103864283* and *LOC103828545*; Tianyou-4: *LOC103828545*), isoamylase (Longyou-7: *LOC103858505* and *LOC103844518*; Tianyou-4: *LOC103858505*), probable sucrose-phosphatase 3a (Longyou-7: *LOC103841339*; Tianyou-4: *LOC103841339*), probable fructokinase-6 (Longyou-7: *LOC103831107*; Tianyou-4: *LOC103831107*), glucan endo-1,3-beta-glucosidase 1 (Longyou-7: *LOC103836214*), beta-glucosidasealpha-glucan phosphorylase (Tianyou-4: *LOC103854138* and *LOC103873279*), hexokinase-3 (Tianyou-4: *LOC103832872*), trehalose-phosphate phosphatase A-like (Tianyou-4: *LOC103833085*), and phosphoglucomutase (Tianyou-4: *LOC103832797*). These results demonstrated that the photosynthesis and carbohydrate metabolism processes might play critical roles in overwintering memory, and that the identified candidate overwintering memory genes provide a new direction for future research on *Brassica rapa* L.

### *WRKY* Transcription Factors May Play an Important Regulatory Role in the Root Development and Winter Memory of *Brassica rapa* L.

Transcription factors (TFs), by binding to specific local and distal cis-elements of a given gene promoter region to regulate its expression, are also critical regulators of various signaling and regulatory networks associated with plant growth and development as well as stress response (Goel et al., 2016; Baillo et al., 2019). In this study, we identified 58 TF families in the three varieties. These include *WRKY, bHLH, C2H2, MYB, ERF, NAC, G2-like, bZIP, HD-ZIP*, etc., which are involved in stress response and viable candidates for crops improvement (Supplementary Figure 6) (Baillo et al., 2019). Among these TFs, the *WRKY* family accounts for the vast majority of all TFs in different stages (Supplementary Table 11), implying the critical role of *WRKY* family members in the root development and overwintering response of *Brassica rapa* L. As one of the 10 largest TF families in plants (Wei et al., 2012), the *WRKY* gene family, out of the numerous TF families, played an important role in various diverse processes such as seed germination (Gu et al., 2017), root growth (Rosado et al., 2021), leaf senescence (Cui et al., 2020), and biotic/abiotic stress responses (Hu et al., 2021; Zhang et al., 2021). In addition, it has been reported that, like lignin, *WRKY* also participates in carbohydrate and secondary metabolites synthesis (Phukan et al., 2016; Jiang et al., 2017; Teng et al., 2021). Apart from that, some previous studies have also postulated the involvement of *WRKY* in plant hormones signal transduction (Dong et al., 2003; Rushton et al., 2012). Zhang et al. (2016) found that *CsWRKY46* can confer cold resistance in transgenic-plant by binding to the W-box in the promoter of ABA-responsive transcription factor *ABI5* to regulate the expression of cold-stress responsive genes. In *Arabidopsis*, transcription factor *AtWRKY46* has been shown to regulate lateral root development by ABA signaling and auxin homeostasis under osmotic/salt stress (Ding et al., 2015). Moreover, Zhu H. et al. (2021) suggested that *AhWRKY75*, by regulating the ROS scavenging system and photosynthesis, confers salt tolerance in transgenic lines of peanut. Furthermore, many *WRKY* TFs can interact with the MAPK signaling pathway (brp04016) to regulate stress response (Yao et al., 2021). Simultaneously, some *WRKY* TFs (*WRKY33*: *LOC103865671, WRKY22*: *LOC103836885, WRKY25*: *LOC103867969, WRKY29*: *LOC103834286*) were also identified in the MAPK signaling pathway in our study. Thus, we speculated that the *WRKY* transcription factor might be an important integrator of phenylpropanoid biosynthesis, plant hormone signal transduction, and the photosynthesis and carbohydrate metabolism processes to regulate the root development and winter memory of *Brassica rapa* L. Molecular orchestration of *WRKY* TFs to combine different pathways would be more interesting to explore in the future.

### A Possible Molecular Regulation Network of Root Development and Overwintering Memory in *Brassica rapa* L.

**Figure 8:**
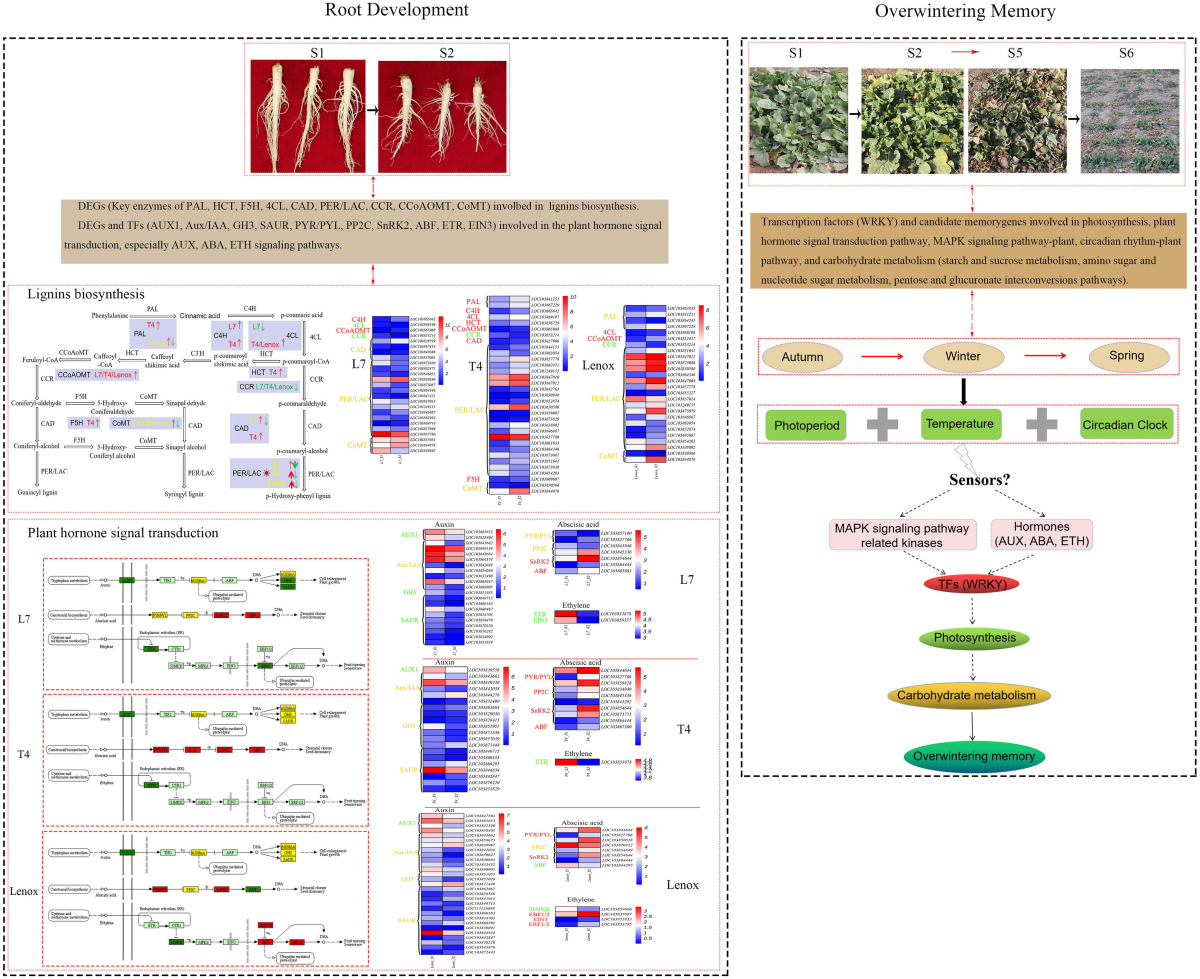
Presumed molecular regulation network underlying root development and overwintering memory in winter rapeseed. The upregulated DEGs annotated in KEGG map and heatmaps were highlighted with red color, the downregulated DEGs were highlighted with green color, and yellow indicates both downregulated and upregulated.

Based on the above results of transcriptomics and previous studies, a possible molecular regulation network underlying the root development and overwintering memory of winter rapeseed was constructed (Figure 8). Briefly, root development of winter rapeseed occurs at an early overwintering period, and can trigger the signal transduction of phenylpropanoid biosynthesis pathway (mainly the lignin biosynthesis branches) and plant hormone signal transduction pathway (especially ETH, AUX, and ABA). Then, for the candidate overwintering memory genes, the memory genes were mainly enriched in the plant hormone signal transduction, MAPK signaling pathway-plant, phenylpropanoid biosynthesis, photosynthesis, carbon fixation in photosynthetic organisms, carbohydrate metabolism (e.g., starch and sucrose metabolism, amino sugar and nucleotide sugar metabolism, and pentose and glucuronate interconversions), and circadian rhythm-plant pathways. During the long overwintering period with fluctuant daily photoperiod and temperature from late autumn to warm spring, some plasma membrane-located sensors lead to changes in plant hormone signal transduction pathways, and then protein kinase genes associated with the MAPK signaling pathway are activated to trigger the expression of downstream genes related to development and response to changed environmental conditions. Hormone signaling and transcription factors (WRKY) also form a complex network to regulate the expression of photosynthesis-related genes. Moreover, the circadian clock may play an important role in transduction of light and temperature signals, and photosynthesis combines with the circadian clock to control starch and sucrose production. These pathways affect carbohydrate metabolism processes and eventually induce metabolic adjustments. Synthesis and storage of some metabolites influence root development and overwintering memory. However, these findings need to be proved by further research. Overall, this study demonstrated that the cold-tolerant variety Longyou-7 might possess a lower degree of cell lignification and a reduced plant hormone signal transduction pathway (e.g., ETH, AUX, and ABA) to accelerate root development in an early overwintering period for successfully living through the winter than the middle cold-tolerant variety Tianyou-4 and the cold-sensitive variety Lenox, which may be the main reason why Longyou-7 has stronger cold resistance than the other two varieties. Furthermore, changes in photosynthesis and carbohydrate metabolism processes may confer Longyou-7 and Tianyou-4 with a yearly “overwintering memory” to distinguish between different seasons and regulate the development and stress response processes.

## Conclusion

In the present study, we conducted transcriptomic analyses to investigate the root development and overwintering memory mechanisms in three winter rapeseed varieties, i.e., Longyou-7 (strong cold tolerance), Tianyou-4 (middle cold tolerance), and Lenox (cold-sensitive). The results showed that the roots of the more tolerant rapeseed variety would develop faster than those of the sensitive variety, and that the differentially expressed genes of the three varieties that were associated with winter rapeseed root development and overwintering memory in an early overwintering period were mainly involved in the phenylpropanoid biosynthesis (particularly of lignin biosynthesis) and plant hormone signal transduction (especially ETH, AUX, and ABA) pathways. Notably, candidate overwintering memory genes related to the photosynthesis and carbohydrate metabolism processes were also identified in Longyou-7 and Tianyou-4. In addition, the *WRKY* transcription factor family may be the important regulators combining different pathways in root development and overwintering memory. Taken together, this study has extended our knowledge of the root development and intricate overwintering memory mechanism of winter rapeseed. Also, it provides essential genetic resources for breeding cold-resistant winter rapeseed varieties. Future studies should characterize the function of memory genes through a series of experiments. We hope that our research will open a new direction to accelerate the resolution of the cold-tolerant molecular mechanism in *Brassica rapa* L.

## Data Availability Statement

The sequenced transcriptome raw data have been deposited to the SRA at NCBI with the accession number of PRJNA811760 (https://www.ncbi.nlm.nih.gov/bioproject/PRJNA811760).

## Author Contributions

LL and WS initiated and designed the study. ZN, FX, LM, and XL performed the experiments. YP, YF, JX, and JY analyzed the data. LL wrote the manuscript. LL and JW provided the funding for this research. All the authors have read and approved the final version of the manuscript.

## Funding

This study was supported by the research program sponsored by the State Key Laboratory of Aridland Crop Science, Gansu Agricultural University, China (No. GSCS2020-08), the National Natural Science Foundation of China (No. 31960435), the Scientific Research Start-up Funds for Openly-recruited Doctors, Science and Technology Innovation Funds of Gansu Agricultural University, China (GAU-KYQD-2018-42), and the China Agriculture Research System of MOF and MARA (CARS-12).

## Conflict of Interest

JX was employed by Shanghai OE Biotech Co., Ltd. The remaining authors declare that the research was conducted in the absence of any commercial or financial relationships that could be construed as a potential conflict of interest.

## Publisher's Note

All claims expressed in this article are solely those of the authors and do not necessarily represent those of their affiliated organizations, or those of the publisher, the editors and the reviewers. Any product that may be evaluated in this article, or claim that may be made by its manufacturer, is not guaranteed or endorsed by the publisher.

## Supplementary Material

Supplementary Figure 1 Correlation analyses of transcriptomes among the three biological replicates of each sampling stage in **(A)** Longyou-7 (L7), **(B)** Tianyou-4 (T4), and **(C)** Lenox. S1–S6, different sampling stages.

Supplementary Figure 2 Statistics of differently expressed genes in different stages of the three varieties.

Supplementary Figure 3 Venn diagrams of differently expressed genes in the S2–S5 stages compared to the S1 stage of Longyou-7 (L7), Tianyou-4 (T4), and Lenox. **(A)** S2 stage, **(B)** S3 stage, **(C)** S4 stage, and **(D)** S5 stage.

Supplementary Figure 4 All expression profiles of DEGs in Longyou-7 in the six sampling stages.

Supplementary Figure 5 All expression profiles of DEGs in Tianyou-4 in the six sampling stages.

Supplementary Figure 6 Top 12 families of differentially expressed transcription factors in different stages of **(A)** Longyou-7 (L7), **(B)** Tianyou-4 (T4), and **(C)** Lenox.

Supplementary Figure 7 Venn diagrams of SNPs, INDELS, and alternative splicing events identified in Longyou-7, Tianyou-4, and Lenox, respectively.

Supplementary Figure 8 qRT-PCR analysis of 11 selected DEGs with different expression patterns in the RNA-seq data in six sampling stages. Histograms represent transcript abundance (FPKM) of the RNA-Seq data (left *y*-axis), and line charts represent the relative expression levels of means ± SD from three independent biological replicates by qRT-PCR (right *y*-axis). The data of the relative expression levels in the S1 stage were set to 1. Correlation between the RNA-Seq and the qRT-PCR of the 11 selected DEGs was also shown.
