# Corrigendum: Transcriptomic insights into root development and overwintering transcriptional memory of *Brassica rapa* L. grown in the field

**DOI:** 10.3389/fpls.2023.1195912

**Published:** 2023-04-12

**Authors:** Lijun Liu, Yuanyuan Pu, Zaoxia Niu, Junyan Wu, Yan Fang, Jun Xu, Fang Xu, Jinli Yue, Li Ma, Xuecai Li, Wancang Sun

**Affiliations:** ^1^ State Key Laboratory of Aridland Crop Science, Gansu Agricultural University, Lanzhou, China; ^2^ College of Agronomy, Gansu Agricultural University, Lanzhou, China; ^3^ Shanghai OE Biotech Co., Ltd., Shanghai, China

**Keywords:** *Brassica rapa* L., transcriptomic, differentially expressed genes, root development, overwintering memory

## Incorrect funding

In the published article, there was an error in the **Funding** statement. The **Funding** statement for the State Key Laboratory of Arid Land Crop Science, Gansu Agricultural University, China was displayed as “No. GSCS-20202-08”. The correct **Funding** statement appears below:

“This study was supported by the research program sponsored by the State Key Laboratory of Aridland Crop Science, Gansu Agricultural University, China (No. GSCS-2020-08).’’

The authors apologize for this error and state that this does not change the scientific conclusions of the article in any way. The original article has been updated.

