# Supplementary figures and images for: Transcriptomic Insights Into Root Development and Overwintering Transcriptional Memory of Brassica rapa L. Grown in the Field
Source: Front Plant Sci. 2022 Jul 22;13:900708. doi: 10.3389/fpls.2022.900708 (PMC9355659; doi:10.3389/fpls.2022.900708)

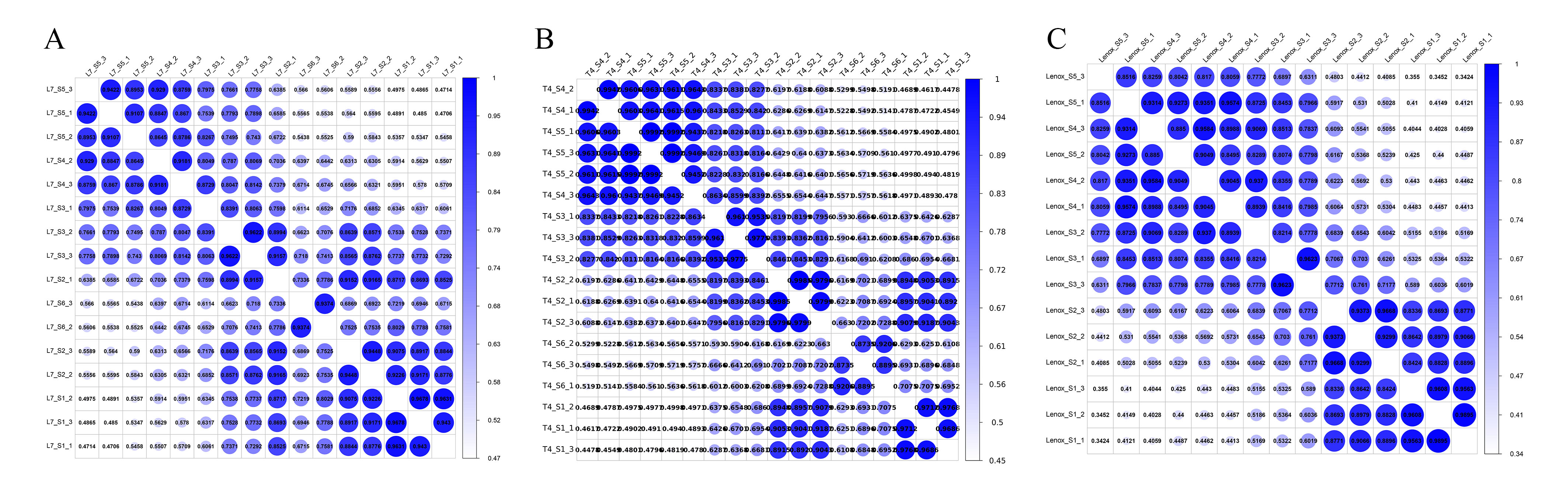

Supplement: Supplementary Figure 1 — Correlation analyses of transcriptomes among the three biological replicates of each sampling stage in (A) Longyou-7 (L7), (B) Tianyou-4 (T4), and (C) Lenox. S1–S6, different sampling stages. [file Image_1.JPEG]

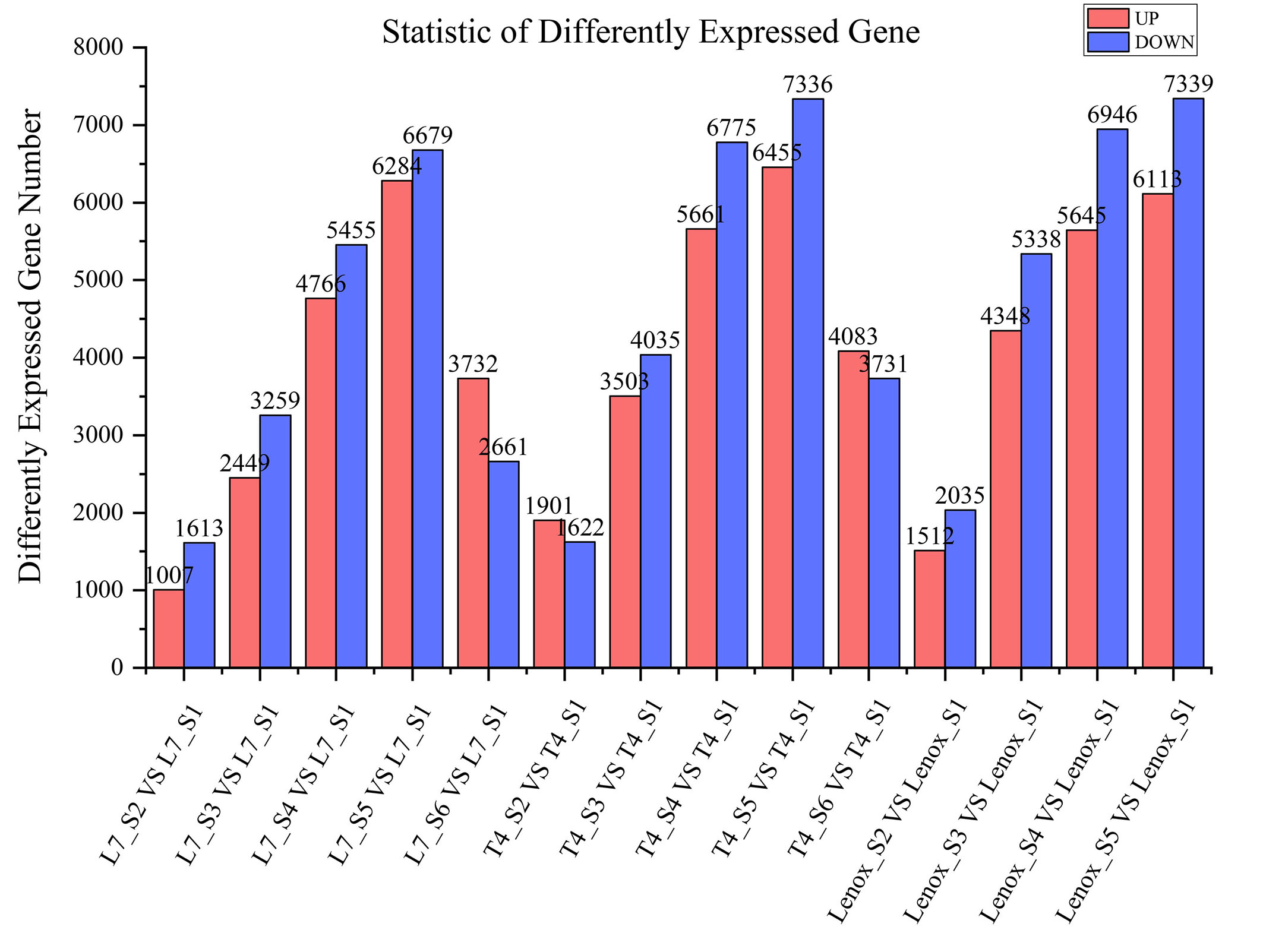

Supplement: Supplementary Figure 2 — Statistics of differently expressed genes in different stages of the three varieties. [file Image_2.JPEG]

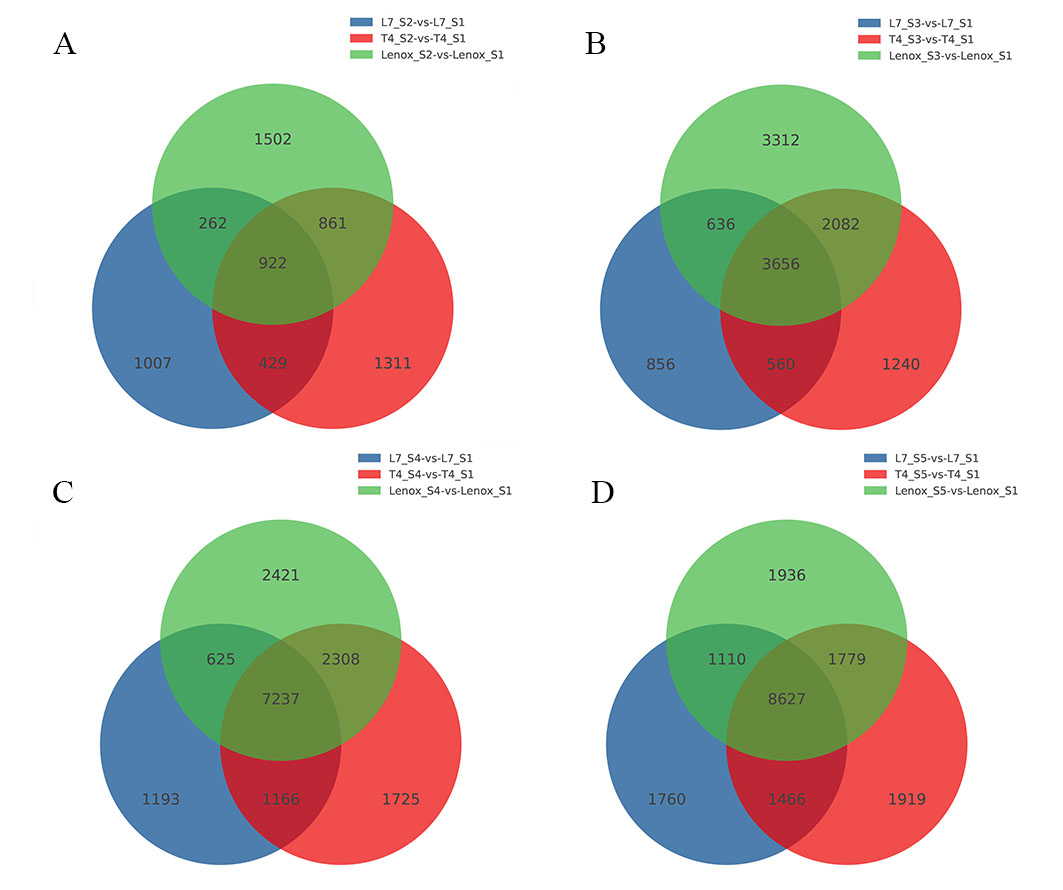

Supplement: Supplementary Figure 3 — Venn diagrams of differently expressed genes in the S2–S5 stages compared to the S1 stage of Longyou-7 (L7), Tianyou-4 (T4), and Lenox. (A) S2 stage, (B) S3 stage, (C) S4 stage, and (D) S5 stage. [file Image_3.JPEG]

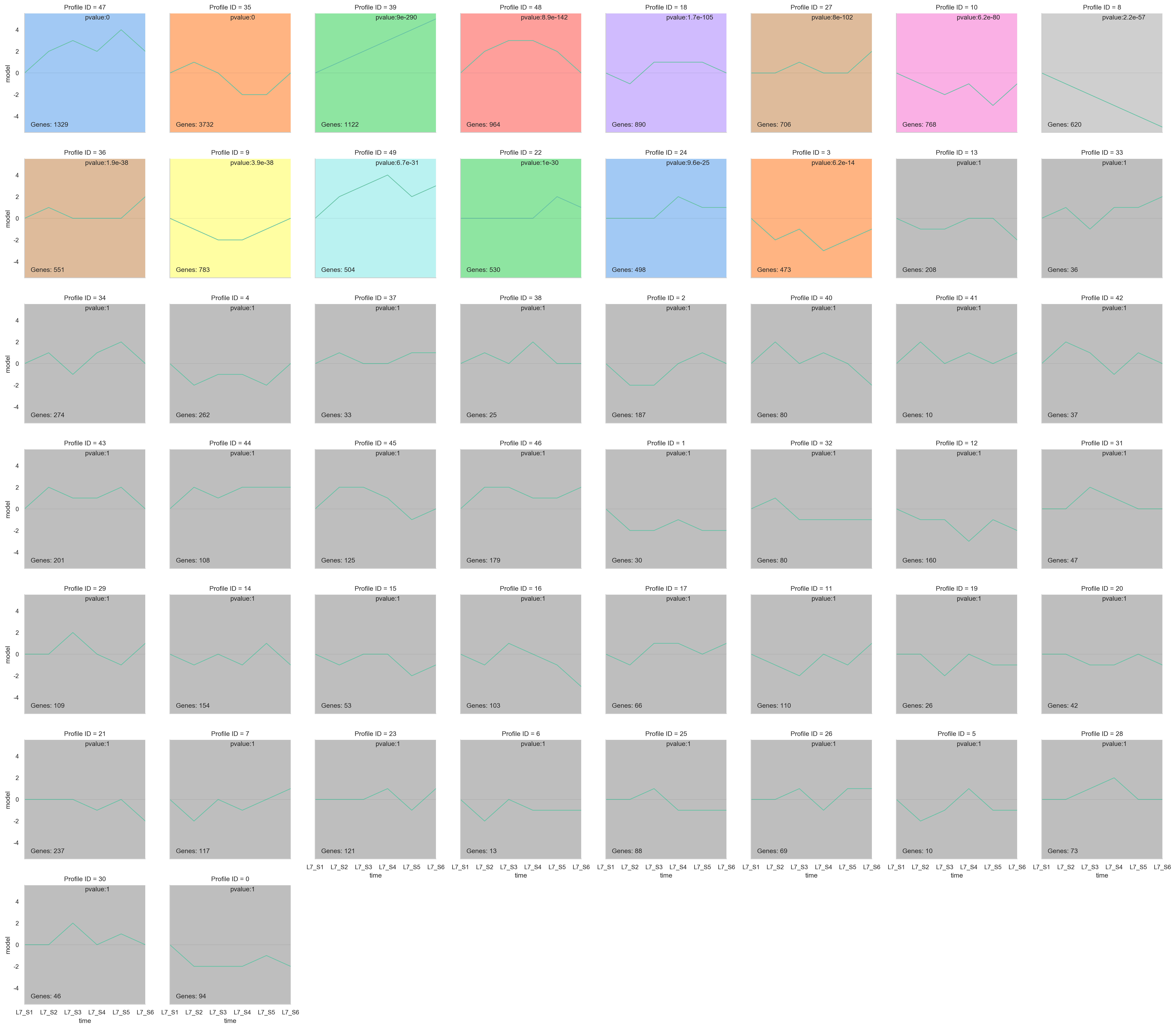

Supplement: Supplementary Figure 4 — All expression profiles of DEGs in Longyou-7 in the six sampling stages. [file Image_4.JPEG]

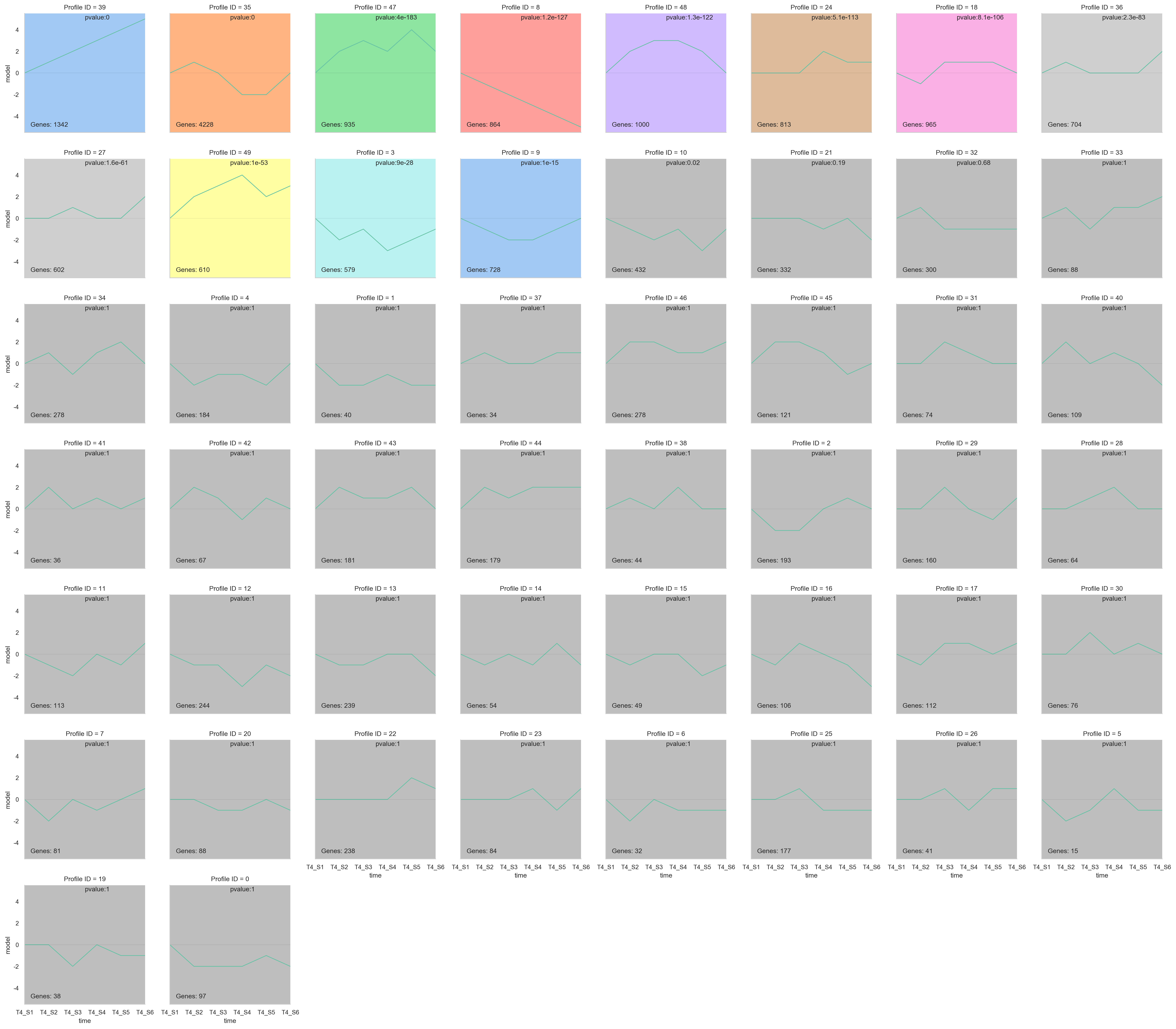

Supplement: Supplementary Figure 5 — All expression profiles of DEGs in Tianyou-4 in the six sampling stages. [file Image_5.JPEG]

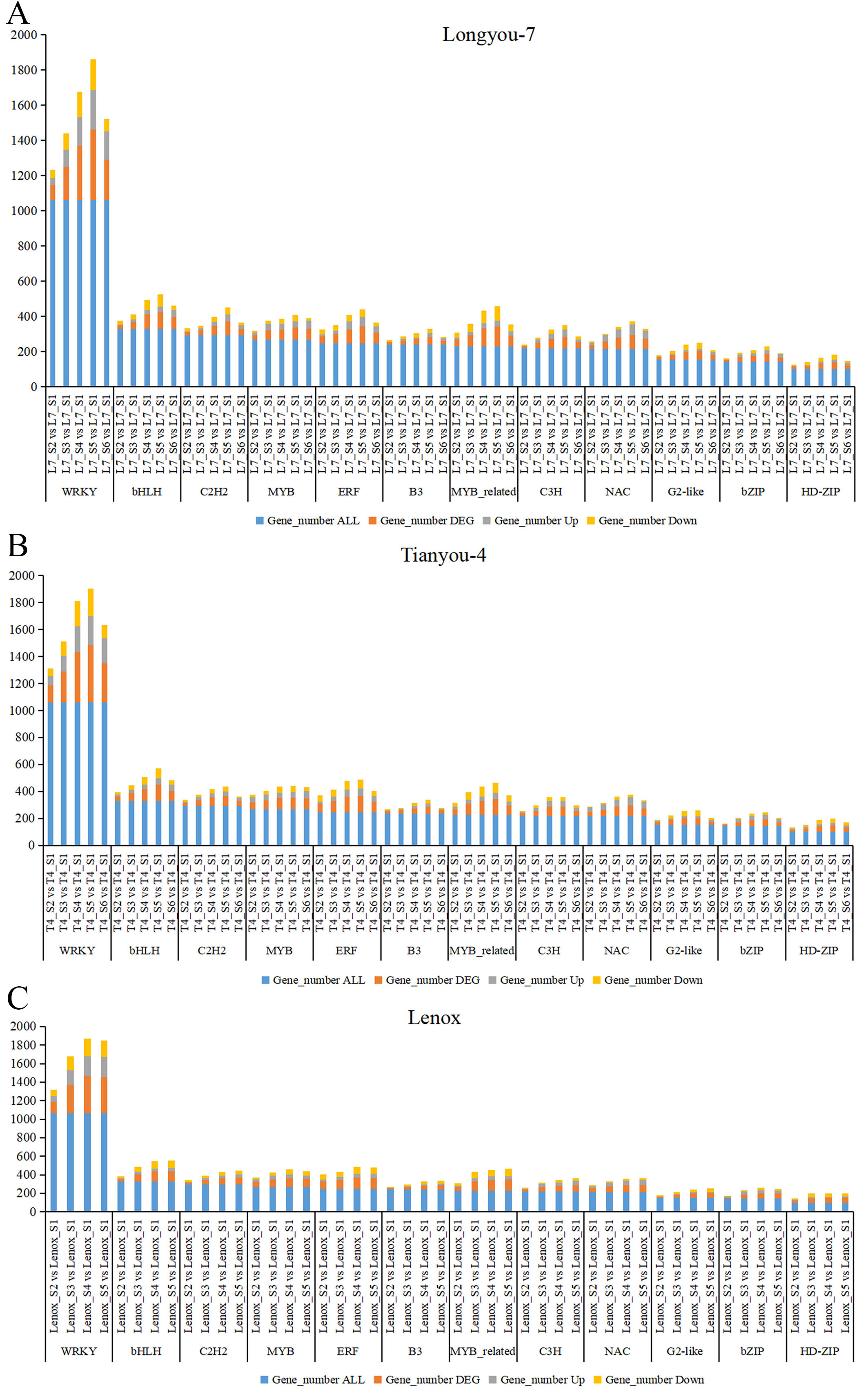

Supplement: Supplementary Figure 6 — Top 12 families of differentially expressed transcription factors in different stages of (A) Longyou-7 (L7), (B) Tianyou-4 (T4), and (C) Lenox. [file Image_6.JPEG]

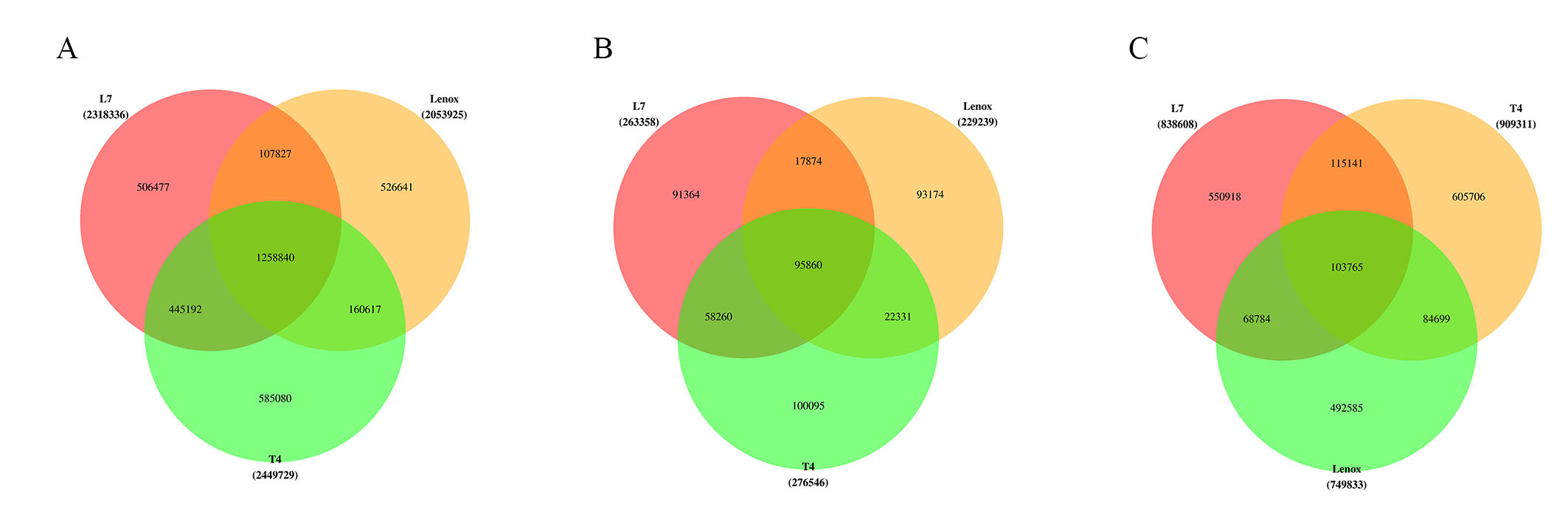

Supplement: Supplementary Figure 7 — Venn diagrams of SNPs, INDELS, and alternative splicing events identified in Longyou-7, Tianyou-4, and Lenox, respectively. [file Image_7.JPEG]

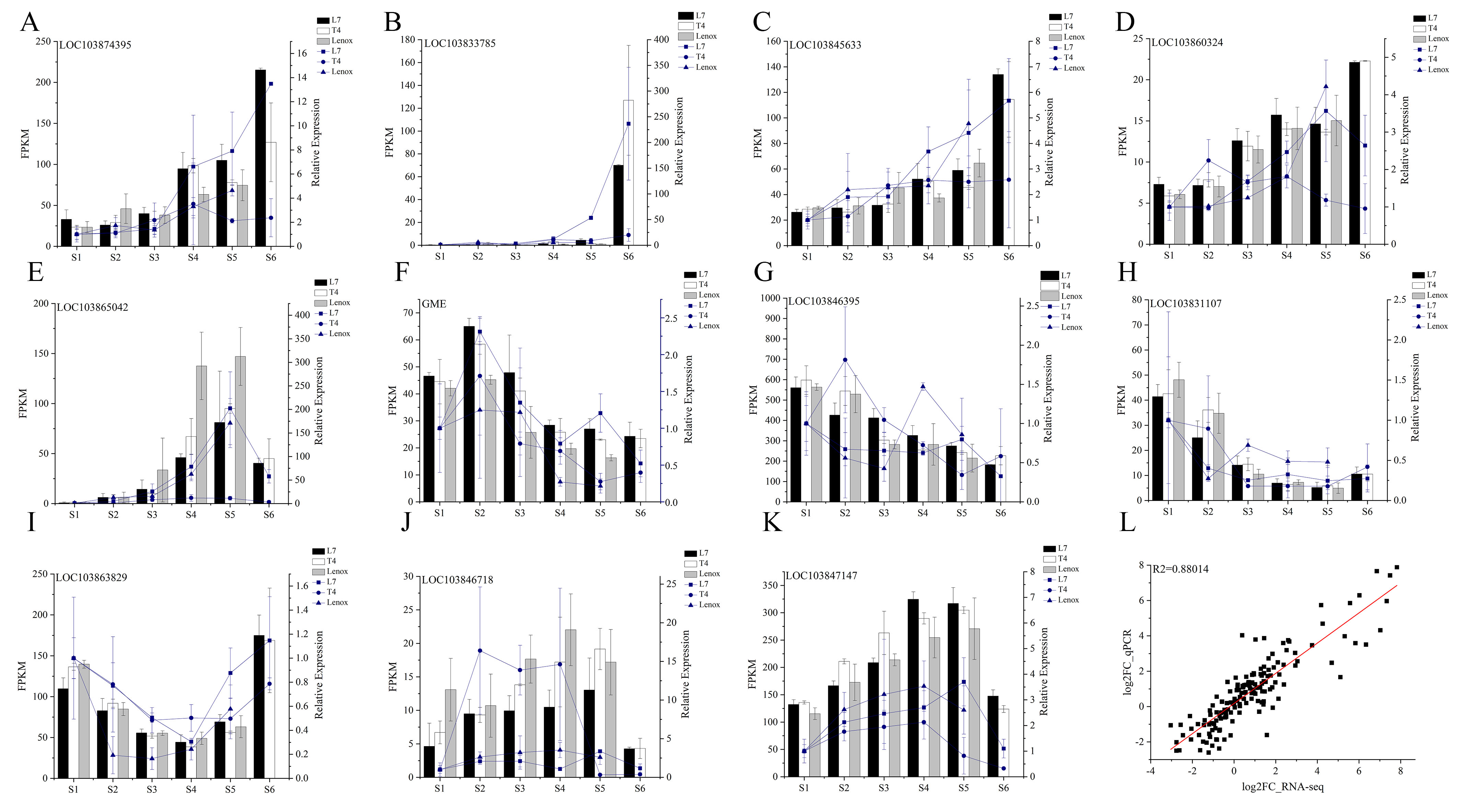

Supplement: Supplementary Figure 8 — qRT-PCR analysis of 11 selected DEGs with different expression patterns in the RNA-seq data in six sampling stages. Histograms represent transcript abundance (FPKM) of the RNA-Seq data (left y-axis), and line charts represent the relative expression levels of means ± SD from three independent biological replicates by qRT-PCR (right y-axis). The data of the relative expression levels in the S1 stage were set to 1. Correlation between the RNA-Seq and the qRT-PCR of the 11 selected DEGs was also shown. [file Image_8.JPEG]
